# Exploring the Phase Space of Multi-Principal-Element Alloys and Predicting the Formation of Bulk Metallic Glasses

**DOI:** 10.3390/e22030292

**Published:** 2020-03-02

**Authors:** Mirko Gabski, Martin Peterlechner, Gerhard Wilde

**Affiliations:** Institute of Materials Physics, University of Münster, Wilhelm-Klemm-Str. 10, 48149 Münster, Germany; matphysik@uni-muenster.de (M.G.); martin.peterlechner@uni-muenster.de (M.P.)

**Keywords:** metallic glasses, glass formation, critical cooling rate

## Abstract

Multi-principal-element alloys share a set of thermodynamic and structural parameters that, in their range of adopted values, correlate to the tendency of the alloys to assume a solid solution, whether as a crystalline or an amorphous phase. Based on empirical correlations, this work presents a computational method for the prediction of possible glass-forming compositions for a chosen alloys system as well as the calculation of their critical cooling rates. The obtained results compare well to experimental data for Pd-Ni-P, micro-alloyed Pd-Ni-P, Cu-Mg-Ca, and Cu-Zr-Ti. Furthermore, a random-number-generator-based algorithm is employed to explore glass-forming candidate alloys with a minimum critical cooling rate, reducing the number of datapoints necessary to find suitable glass-forming compositions. A comparison with experimental results for the quaternary Ti-Zr-Cu-Ni system shows a promising overlap of calculation and experiment, implying that it is a reasonable method to find candidates for glass-forming alloys with a sufficiently low critical cooling rate to allow the formation of bulk metallic glasses.

## 1. Introduction

While conventional alloys such as steel (>80 at% Fe, <2 at% C, and 1–5 at% of Co, Mo, V, and W) usually consist of only one principal element and several minor alloying elements, multi-principal-element alloys (MPEAs) consist of, as the name suggests, multiple constituents in a comparable concentration. Well-known examples are the Co_20_Cr_20_Fe_20_Mn_20_Ni_20_ high entropy alloy [1], and the so-called Vitreloys (e.g., Vitreloy 1 Zr_41.2_Be_22.5_Ti_13.8_Cu_12.5_Ni_10_), a group of commercially used metallic glasses [2,3].

Seeing that MPEAs can be either glasses, high entropy alloys or, in the case of high entropy metallic glasses, both at the same time [4], it is reasonable to assume that there are some shared concepts or parameters whose values and exact behavior result in the formation of either amorphous metallic glasses or crystalline solid solution phases. This idea was proposed by Zhang et al. for high entropy alloys due to the observation that certain ranges of atomic size mismatch between the alloyed elements and a certain interval of the entropies of mixing which, at the melting temperature, exceed the enthalpy of mixing, lead to the formation of solid solution phases [5]. Guo and Liu observed that for MPEAs there is a correlation between a set of parameters consisting of the mismatch in atomic radii *δ*, the enthalpy of mixing ∆*H*_mix_ and the entropy of mixing ∆*S*_mix_ and the tendency to form a solid solution or amorphous phases [6].

## 2. Materials and Methods 

In the following we expand this approach, utilizing the following definitions [5,6]:

(1) $$\Delta S_{\mathrm{mix}}=-R\sum_{i=1}^{N}c_{i}\ln c_{i},$$

(2) $$∆H_{\mathrm{mix}}=\sum_{i=1,\;i\neq j}^{N}4H_{ij}^{\mathrm{mix}}c_{i}c_{j},$$

(3) $$T_{m}=\sum_{i=1}^{N}c_{i}T_{m,i},$$

(4) $$\Omega =\frac{T_{m}\Delta S_{\mathrm{mix}}}{\left|∆H_{\mathrm{mix}}\right|},$$

(5) $$\delta =\sqrt{\sum_{i=1}^{N}c_{i}{\left(1-\frac{r_{i}}{\bar{r}}\right)}^{2}},$$

(6) $$\bar{r}=\sum_{i=1}^{N}c_{i}r_{i},$$

where $\Delta S_{\mathrm{mix}}$ is the configurational entropy of mixing in J·K^−1^·mol^−1^, R the universal gas constant, $c_{i}$ the atomic fraction of element i, $∆H_{\mathrm{mix}}$ the enthalpy of mixing in kJ·mol^−1^, $H_{ij}^{\mathrm{mix}}$ the enthalpy of mixing of the binary alloy consisting of element i and j, $T_{m}$ the averaged melting temperature, Ω is a dimensionless parameter, given by the ratio of the product of melting temperature and entropy of mixing $T_{m}\Delta S_{\mathrm{mix}}$ (in K·(J·K^−1^·mol^−1^)) and the enthalpy of mixing $∆H_{\mathrm{mix}}$ (in kJ·mol^−1^), and δ the difference of atomic radii $r_{i}$ from the mean atomic radius $\bar{r}$. Guo and Liu concluded the following ranges for the formation of solid solution (SS) and amorphous phases (AM) by correlating the experimentally observed phases with these calculated parameters [6]:

Solid solution phases: 

−22 kJ·mol^−1^  ≤ $∆H_{\mathrm{mix}}$ ≤ 7 kJ·mol^−1^

11 J·K^−1^·mol^−1^ ≤ $\Delta S_{\mathrm{mix}}$   ≤ 19.5 J·K^−1^·mol^−1^

     0% ≤ *δ*     ≤ 8.5%

Amorphous phases: 

−35 kJ·mol^−1^   ≤ $∆H_{\mathrm{mix}}$ ≤ −8.5 kJ·mol^−1^

7 J·K^−1^·mol^−1^ ≤ $\Delta S_{\mathrm{mix}}$   ≤ 14 J·K^−1^·mol^−1^

      9% ≤ *δ*

The parameter Ω given by the ratio $\Omega \;=\;T_{m}\Delta S_{\mathrm{mix}}/\left|∆H_{\mathrm{mix}}\right|$ amounts to Ω≥1.1 for solid solution high entropy alloys, indicating that at the melting temperature the effect of the entropy of mixing surpasses the effect of the enthalpy of mixing leading to the formation of a solid solution phase [7]. The simplicity of the calculation lends itself to the quick exploration of the phase formation behavior of varying alloy compositions based on the given constraints by generating all possible compositions for a chosen alloy. It is furthermore reasonable to calculate the critical cooling rate $R_{C}$ for the case of amorphous or metallic glass phases to estimate the glass-forming ability of an alloy not only based on thermodynamic but also kinetic arguments. According to the empirical model proposed by Takeuchi and Inoue, the critical cooling rate $R_{C}$ can be calculated as [8]:

(7) $$R_{C}=Z\frac{k_{B}T_{m}^{2}}{a^{3}\eta_{T=T_{m}}}\exp\left(\frac{∆G}{RT}\right),$$

where $Z\;=\;2\;\times \;10^{-6}$ is a constant obtained from the analysis of sodium chloride, $k_{B}$ is the Boltzmann constant, $T_{m}$ is the melting temperature, a is the lattice constant, and $\eta_{T=T_{m}}$ is the viscosity at the melting temperature, which itself can be expressed using Andrade’s equation [9]:

(8) η(Tm)=CAATmV23,

where $C_{A}$ is a constant of $1.85\times 10^{-7}$ (J∙K^−1^∙mol^⅓^) ^½^ adopted for glass-forming systems, A is the atomic weight, and V the molar volume. Here the atomic volume and mass are averaged analogous to the averaged atomic radii or melting temperatures. The lattice constants of the melts were estimated from the atomic radii by assuming that a typical molten metal has an average coordination number of 8.9 [10].

∆G at T is taken as $∆G\;=\;∆H_{\mathrm{mix}}-T\Delta S_{\mathrm{mix}}$.

Atomic radii $r_{i}$ were taken from reference [6], mixing enthalpies $∆H_{\mathrm{mix}}$ were taken from reference [11], atomic weights A, volumes V, and melting temperatures $T_{m}$ were taken from reference [12]. This set of parameters coincides with a set of assumptions made for the used model: $∆H_{\mathrm{mix}}$ is calculated according to the regular melt model, $\Delta S_{\mathrm{mix}}$ is calculated as that of a regular solution with N-elements, the calculation of δ assumes that the different species of atoms are homogenously and randomly distributed in both amorphous and solid solution phases [5], and the melting temperatures $T_{m}$ are estimated using the rule of mixing to keep the involved calculation simple. Furthermore, it is assumed that the alloys investigated by Guo and Liu are representative, and the given parameter ranges are therefore valid for a wide range of alloys.

**Figure 1:**
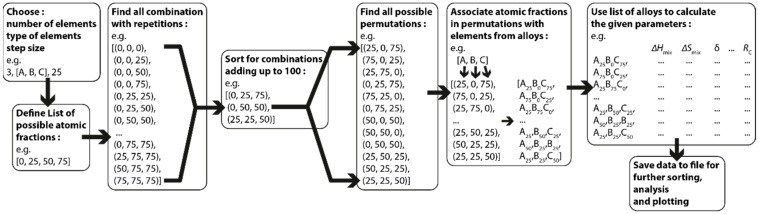
Flowchart depicting the process of generating alloy compositions for the calculation of their thermodynamic and kinetic parameters.

To generate all possible compositions for a given alloy system a simple combinatorics-based approach, using the “itertools” module for python 3, was used to keep the computation times and memory consistent and predictable, and to avoid deep recursions or potentially infinite loops. The used combinatorics and calculations are schematically shown in Figure 1 and will be explained in the following paragraph. First the number of components N, the elements of interest, and the step-size for the atomic fractions are chosen. Based on the step size, a list of all possible values for the atomic fractions is created. From this list of possible values, a list of all combinations of N atomic fractions is generated while combinations not adding up to 100 at% are discarded afterwards. In the next step all permutations of each tuple of N atomic fractions are generated and each tuple index is associated with one of the chosen elements resulting in a list of all possible alloys for the given elements with the desired step size. Subsequently the parameters defined in Equations (1)–(7) are then calculated for all alloys in the generated list.

Generally, the here developed approach and python scripts can serve to find e.g.,:

- A composition range of already known elements with best glass-forming ability;
- Additional alloying elements for a known basic alloy to form a glass;
- Any glass-forming alloy (limited by the database of elements).

A point-by-point description on the applications of the python scripts can be found in the Supplementary Material, including the scripts and an introductory PDF file.

## 3. Results

In the following the computed results are evaluated and compared to experimental data. First, the run-time using a conventional desktop PC (in 2020) is tested, see Section 3.1. Run-time. Then, some results from main applications for the scripts are presented: 3.2. Glass-Formation: The analysis of the glass-forming ability of an alloy with known elements, 3.3. Micro-Alloying: The search for additional elements to improve the glass-forming ability, and finally, in 3.4. Search for New Glasses: The search for any combination of elements forming a glass. 

### 3.1. Run-Time

**Figure 2:**
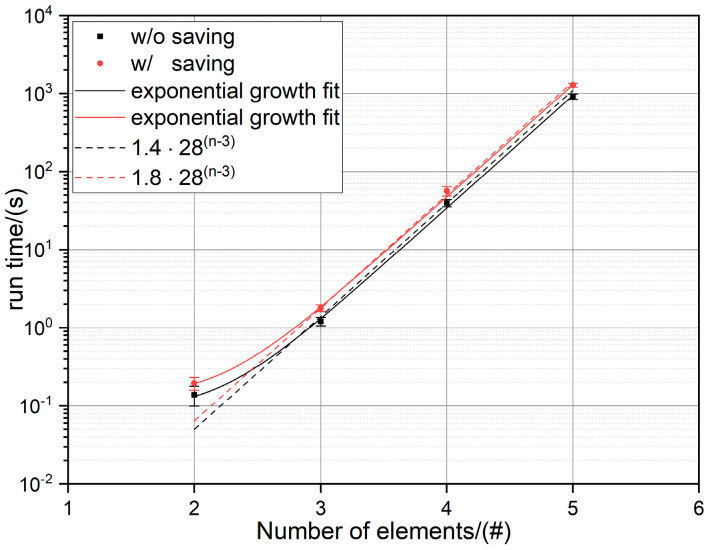
Flowchart depicting the process of generating alloy compositions for the calculation of their thermodynamic and kinetic parameters.

The run time for 2 to 5 component alloys with a step size of 1 at% was recorded for 5 runs each, both including the time for writing the data to the hard drive and excluding it (see Figure 2). All tests were performed using an Intel^®^ Core™ i3-3220 with 2 x 3.30 GHz and 8 GB of RAM running on a 64-bit Windows 10 operating system. Based on an exponential fit one can assume for N≥5 that the run time $t_{N}$ follows the following estimator function:

(9) $$t_{N}=C\cdot 28^{N-3}\;s,$$

where C = 1.4 when excluding and C = 1.8 when including the time for saving the calculated parameters to a data file. From the estimator function one can estimate the run times for a 6 and 7 component system to 9 h and 10 days, respectively. The long runtime can be circumvented by increasing the step size. For example, increasing the step size from 1 to 2 at% decreases the run time for a 5-component system by roughly a factor of 10 from 15 min down to about 90 s.

### 3.2. Glass-Formation

**Figure 3:**
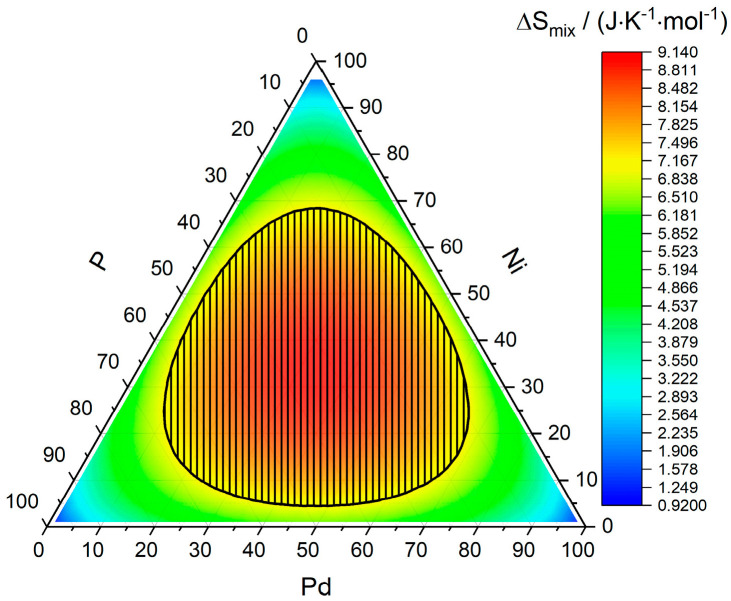
Entropy of mixing of the Pd-Ni-P system. Representative for any 3-component alloy under the employed model. Area that fulfills 7 J·K^−1^·mol^−1^≤ $\Delta S_{\mathrm{mix}}\;$≤ 14 J·K^−1^·mol^−1^ is shaded in black.

**Figure 4:**
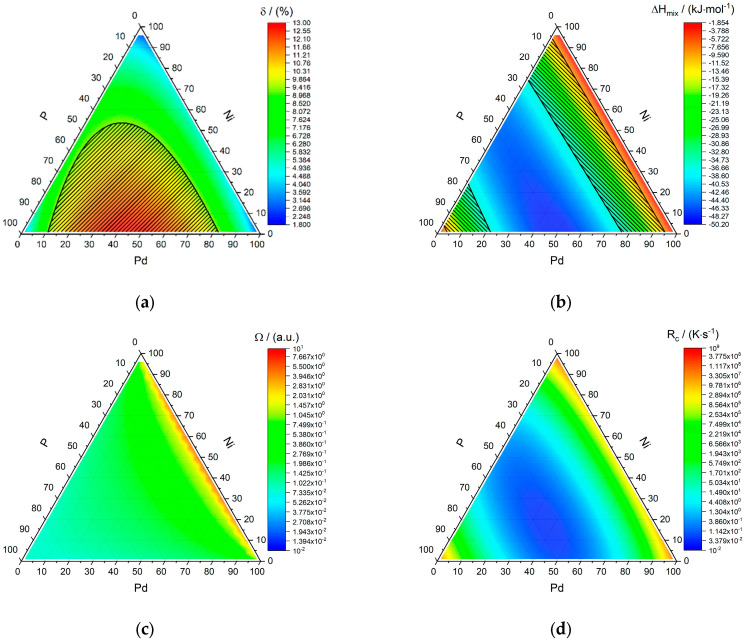
Overview of calculated parameters of the Pd-Ni-P system. Difference in atomic radii *δ* (a, area fulfilling 9% ≤ *δ* is shaded in black (**a**). Top right: Enthalpy of mixing $∆H_{\mathrm{mix}}$, area fulfilling −35 kJ·mol^−1^ ≤ $∆H_{\mathrm{mix}}\;$ ≤ −8.5 kJ·mol^−1^ is shaded in black (**b**). Bottom left: Ratio of the enthalpy of mixing and the entropy of mixing at the melting temperature Ω (**c**). Bottom right: Critical cooling rate $R_{C}$ (**d**).

The famous Pd-Ni-P system shall be used as an example system to showcase the obtained values. Figure 3 shows the entropy of mixing for the 3-component system Pd-Ni-P, although it stands as a representative for any 3-component system as the entropy of mixing does not depend on the type of elements used, but only on their number and atomic fractions. It is important to keep in mind that the entropy of mixing used in this model is only the statistical entropy of mixing obtained for a random solution, thereby ignoring other entropy contribution and assuming that the vibrational entropies of the crystalline and glassy phases are similar in value. The yellow to red center region corresponds here to the range of entropies of mixing of amorphous phases given by Guo and Liu [6]. δ, $∆H_{\mathrm{mix}}$, Ω, and $R_{C}$ are shown in Figure 4. The most negative enthalpy of mixing is achieved along the line of 50 at% of phosphorous, which corresponds to phosphides ranging from pure NiP over (Pd_x_Ni_1−x_)P to PdP. The regions matching the criterion proposed by Guo and Liu [6] coincide with either a very high phosphorous content ranging from 75% to 95% or lower phosphorous contents ranging from 5% to 25%, the latter being the one most Pd-Ni-P bulk metallic glasses (BMGs) fall into [13,14,15,16,17,18]. For all compositions, the condition Ω≤1 holds, meaning that regardless of the chosen composition, the effect of the enthalpy of mixing surpasses that of the mixing entropy at the melting temperature. The critical cooling rates RC along the line of 20 at% phosphorous yield values of ~10 to ~100 K/s (7.1 K/s for Pd_40_Ni_40_P_20_), which is an excellent estimate in light of the simplicity of the used model and compares well to the experimentally obtained value of $R_{C}$ = (35.0 ± 0.5) K∙s^−1^ for Pd_40_Ni_40_P_20_ [19], as well as the observation that Pd_40_Ni_40_P_20_ can be reproducibly cast as a glass with cooling rates as low as 1.4 K∙s^−1^ without and 0.6 K∙s^−1^ with boron trioxide fluxing [20,21,22].

**Figure 5:**
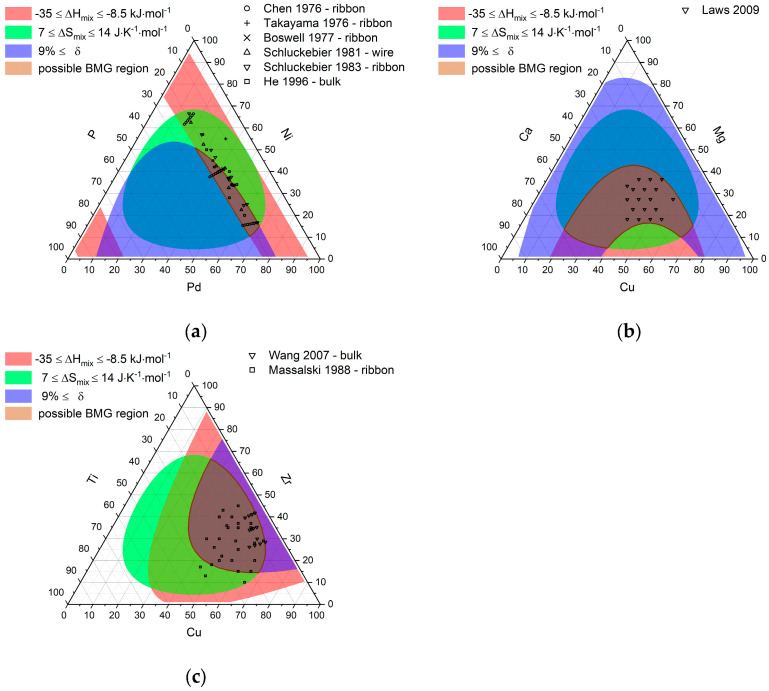
Areas where the conditions for BMG formation are fulfilled for Pd-Ni-P (**a**), Cu-Mg-Ca (**b**), and Cu-Zr-Ti (**c**). Possible region is highlighted in brown. Experimental data taken from references [13,14,15,16,17,18] for Pd-Ni-P, [25] for Cu-Mg-Ca, and [26,27] for Cu-Zr-Ti.

To validate the usefulness of the calculated parameter and verify the parameter ranges given by Guo and Liu [6], the possible bulk metallic glasses forming regions for the systems Pd-Ni-P, Cu-Mg-Ca, and Cu-Zr-Ti were determined by finding the compositions in which all three conditions are met simultaneously. Figure 5 depicts the ternary phase triangles for the three investigated systems, where the compositions with: −35 kJ·mol^−1^ ≤ $∆H_{\mathrm{mix}}\;$≤ −8.5 kJ·mol^−1^ are shaded in red, with 7 J·K^−1^·mol^−1^≤ $\Delta S_{\mathrm{mix}}\;$≤ 14 J·K^−1^·mol^−1^ shaded in green, and with δ≤9% shaded in blue. The possible glass-forming regions are determined by the intersection of the former and are shaded in brown. Experimentally confirmed glasses are noted by symbols for the evaluated systems. For Pd-Ni-P, six out of 10 BMGs fall into the predicted region while 30 of 58 ribbons (or in one case wires) fall into it. By lowering the limit for the mismatch of the atomic radii to δ=8.5%, this can be increased to nine out of 10 bulk glasses and 46 out of 58 ribbons and as such encompassing most of the glass-forming Pd-Ni-P alloys. The observation that some Pd-Ni-P ribbons and some of the bulk metallic glasses lie outside of the predicted region is easily explained by the good glass-forming ability of the Pd-Ni-P system and the fact that the correlation of the used approximation for the viscosity is only good for marginal glass formers and less appropriate for good glass formers, leading to larger deviations from experimentally expected values [9]. When comparing the possible bulk glass-forming regions with the calculated critical cooling rates it is evident that all ribbons show a critical cooling rate below the 104 K∙s^−1^ lower estimate for achievable cooling rates during melt spinning and that all ribbons fall into the range of critical cooling rates with typical values of 10 to 100 K∙s^−1^ achievable during bulk casting [23,24]. All experimentally confirmed bulk glasses for the Cu-Mg-Ca system fall into the predicted region, while all but one out of 13 bulk metallic glasses and 24 out of 27 ribbons align with the prediction for the Cu-Zr-Ti system.

The uncertainties of the calculations are estimated from a simple propagation of uncertainties starting from the input values. All physical constants are assumed to be exact due to their high accuracies or exact definitions. Considering typical sample masses for bulk metallic glasses (few grams) and accuracies for laboratory scales (10^-2^ mg) the uncertainties of the atomic fractions ci can be assumed to be in the range of 0.01%. The uncertainties for the other parameters can be found in the corresponding references [6,11,12]. Estimates for the uncertainties of the calculated parameters are 1.5 J·K^−1^·mol^−1^ for $\Delta S_{\mathrm{mix}}$, 3.5 kJ∙mol^−1^ for $∆H_{\mathrm{mix}}$, 10 K for $T_{m}$, and 0.3 % for δ in absolute values, and 1% for Ω and 160% for $R_{C}$ as relative values, due to their wide range in magnitudes compared to the other four parameters.

### 3.3. Micro-Alloying

**Figure 6:**
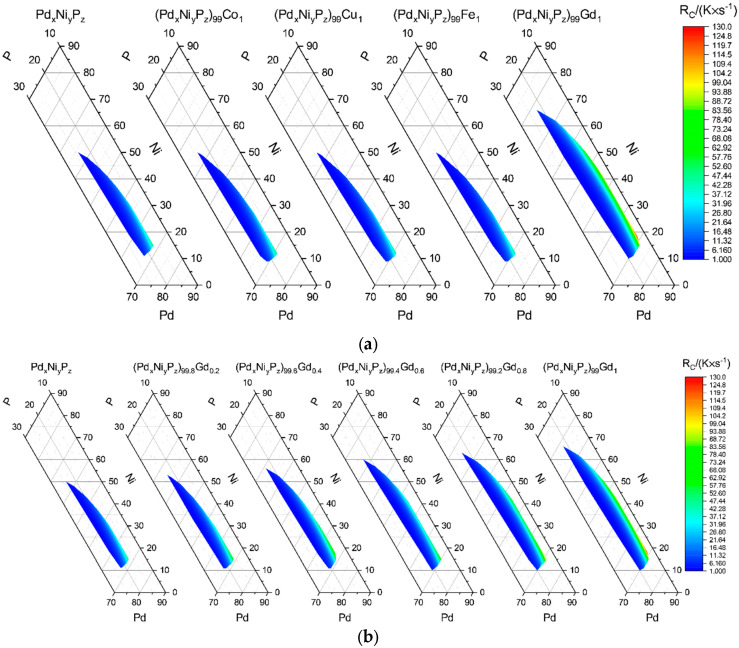
Effects of micro-alloying on the critical cooling rate and predicted bulk metallic glass formation region for different micro-alloyed elements (**a**) and for increasing concentrations of gadolinium (**b**).

To investigate the impact of small concentrations of an additionally added element on a glass-forming alloy, the critical cooling rates and glass formation regions are computed for the Pd-Ni-P-system micro-alloyed with 1 at% of Co, Cu, Fe, and Gd. Micro-alloying of Co, Cu, and Fe had little effect on the overall glass-forming ability which matches well with experimental results for (Pd_40_Ni_40_P_20_)_99_Co_1_ and (Pd_40_Ni_40_P_20_)_99.4_Fe_0.6_ that showed an impact on the mechanical properties without affecting the glass formation properties in a significant way (see Figure 6) [28,29]. 

This behavior is to be expected within the framework of the current model since cobalt, copper, and iron are similar in atomic weight and radius to nickel and therefore will not impact the calculation. This is different for gadolinium, since it varies in its properties significantly from the former alloying elements. The impact of the micro-alloying of gadolinium is even noticeable for concentrations below 1 at% as shown in Figure 6 within the employed model.

### 3.4. Search for New Glasses

Seeing that the used approach so far yielded a good match of calculated possible metallic glasses and experimentally known glass formers it is reasonable to extrapolate from a modified version of this approach to search for new metallic glasses. The simplest approach would be to generate alloys at random, calculate the parameters, and check if they fulfill the proposed conditions, which can serve as a tool to inspire the avid experimenter with new glass-forming compositions formerly not thought about. Using this entirely random approach it is possible to find 100, 1000, and 10,000 compositions which fulfill the given parameters in (0.557 ± 0.026) s, (6.170 ± 0.529) s, and (59.992 ± 4.291) s, respectively (averaged over 10 runs each), with calculated critical cooling rates $R_{C}$ ranging from 10^−3^ K∙s^−1^ to 10^6^ K∙s^−1^ and thereby exceeding the typical cooling rates needed for the formation of bulk metallic glasses by several orders of magnitude. Glasses found in this manner can furthermore be used as starting point to explore the glass-forming regions for alloys with more than five components to constrain the possible space of composition only to the glass-forming ones. This is done by varying the composition of a found possible glass former by a small randomized amount and checking if the conditions of glass formation are met by the newly found alloy. If the new composition lies within the possible glass-forming region it is accepted and used as the starting point for a new random step, otherwise it is discarded and a new step is taken from the previous composition (“find and vary”-method). Glass-forming regions found using this approach exactly match the regions found for the three-component-systems already discussed before and should thereby be a viable approach for alloys with a higher number of components.

**Figure 7:**
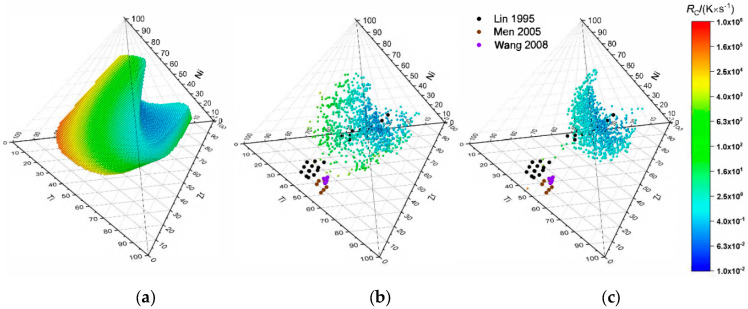
Calculated $R_{C}$ for the Ti-Zr-Cu-Ni system depicted by 3D spheres: Calculated for the entire phase space and filtered for the possible glass-forming region resulting in 70,803 data points (**a**), calculated for 1000 points using the “find and vary”-method (**b**), and the “minimize $R_{C}$ “-method (**c**). Colored 2D circles depict experimentally confirmed BMGs [30,31,32].

To enhance the chances of successfully identifying a new BMG, it is conducive to bias this randomized approach toward lower critical cooling rates. This is achieved by checking if the newly found alloy is a possible glass-forming candidate and also shows a lower $R_{C}$ than the previously found alloy. To avoid being trapped in a local minimum in $R_{C}$ each step has a chance to accept a higher value for $R_{C}$ to allow the algorithm to escape local minima. This method shall be called “minimize $R_{C}$”-method in the following text. Values for $R_{C}$ are accepted if a randomly generated number x∈[0,1] is smaller than the value of $\frac{1}{2}\left[1+\mathrm{erf}\left(\frac{∆R_{C}-\mu }{\sigma }\right)\right]$ where $∆R_{C}$ is the difference in critical cooling rate in-between two steps and μ and σ are expected value and variance of the error function. μ and σ can then be used to tune the probability that a step towards higher critical cooling rates is accepted. For the sake of presentability the four-component system Ti-Zr-Cu-Ni is used to compare the three different computational methods. A sample of 1000 possible glass-forming alloys found using the “find and vary”- and “minimize $R_{C}$”-methods are shown in Figure 7b,c. When compared to the calculation of $R_{C}$ for the entire phase space (see Figure 7a) it is evident that the “find and vary”- method resembles the shape found by the complete calculation sufficiently to obtain a reasonable estimate of the glass-forming region while needing considerably less points. The same holds true for the “minimize $R_{C}$”-method while it maps the region close to the minimal critical cooling rate in more detail. The calculated region encompasses some but not all of the experimentally found BMGs, but it is reassuring to see that experimentally verified glasses exist close to the composition of the calculated minimum of $R_{C}$.

Seeing that the calculation of “phase”-triangles and tetrahedrons for known systems provides good results, we propose the use of the “minimize $R_{C}$”-method for randomly chosen glass-forming alloy systems to find the composition that yields the minimum critical cooling rate. The presented function requires only the number of elements as an input parameter, but allows the specification of one or more elements or even a known starting composition. This allows one to minimize the critical cooling rate for a system based on known glass formers as well as the calculation of completely new glass-forming candidates (see Tables S1 and S2 for possible glass-forming candidates calculated this way). 

## 4. Discussion

The computation times to calculate all compositions for alloys with up to six constituents and check for glass-forming compositions are nine hours or below and therefore lend themselves well to explore alloy system of interest for potential metallic glass candidates. For the presented example of Pd-Ni-P 60% of bulk glasses and 52% of ribbons fall into the prediction for the given parameter ranges and 90% of bulk glasses and 79% of all ribbons, when the cut off for the atomic size mismatch is lowered to δ ≥ 8.5. The experimentally found glasses furthermore spanned over most of the predicted area and were only found outside this area only for metallic glass ribbons. For Cu-Mg-Ca all bulk metallic glasses fell into the predicted region and spanned about half of the predicted region. Here the calculated critical cooling rates covered a rather broad range from $2.5\cdot 10^{4}$ K∙s^−1^ to $2\cdot 10^{5}$ K∙s^−1^, which could be explained by the deviation of around 500 K in calculated melting temperatures compared to the measured melting temperatures presented by Laws et al. [25]. Using the sample dimension-based estimate for the critical cooling rate shown in the work of Lin and Johnson one would estimate cooling rates below 500 K∙s^−1^ for the achieved sample diameters in the work of Laws et al. [25,30]. For Cu-Zr-Ti, 92% of the bulk metallic glasses fell into the predicted region but they only spanned a small fraction of it. This could be due to the fact that the used approach overestimated the number of possible glass-forming composition, although for the case of Pd-Ni-P it seemed that the number of possible glass-forming compositions was underestimated rather than overestimated. The second explanation might be that the shown bulk metallic glasses for the Cu-Zr-Ti system were variations of the well-researched Cu_60_Zr_30_Ti_10_ alloy [33,34,35,36,37,38,39,40]. Ribbons for the Cu-Zr-Ti system spanned a much bigger area of the predicted region, especially toward higher critical cooling rates. The calculated critical cooling rates for the predicted glass-forming compositions for the Cu-Zr-Ti system ranged from $5\cdot 10^{2}$ K∙s^−1^ for Cu_45_Zr_47_Ti_8_ up to $2\cdot 10^{4}$ K∙s^−1^ for Cu_68_Zr_15_Ti_17_ with 1073 out of 1100 candidates below $10^{4}$ K∙s^−1^ and 392 below $10^{3}$ K∙s^−1^, aligning with the regions of possible bulk glasses and ribbons, respectively. Overall it is expected for the used model to deviate more for system with deep eutectics. The calculated critical cooling rates for the quaternary Ti-Zr-Cu-Ni system ranged from $10^{-2}$ K∙s^−1^ to $3\cdot 10^{4}$ K∙s^−1^, thereby reaching values that were four orders of magnitude lower than the lowest cooling rates achieved by the ternary Cu-Zr-Ti system, which aligns with the estimated difference in critical cooling rate given by Lin and Johnson, when comparing the ternary system ($5\cdot 10^{5}$ K∙s^−1^) to their best performing quaternary glass ($2.5\cdot 10^{2}$ K∙s^−1^), which could be cast at a thickness of 4 mm without crystallization [30]. When comparing the results shown in Figure 7 for the different algorithms it is obvious that a good approximation of the overall shape of the complete glass-forming region with 70,803 data points can be achieved with considerably less data points, in this case 1000, using the described “find and vary”-method. This leads to a considerable decrease in computation time for the intended purpose of finding promising bulk metallic glass candidates. While the use of the “minimize $R_{C}$.”-method” more accurately depicts the region around the minimum critical cooling rate and is therefore a good candidate for a method to compute suitable glass-forming candidates for a given or randomly selected alloy. It should be noted however that the assumptions underlying the presented approach impose some limitations on the model. Firstly, the used definition for the average melting temperature $T_{m}$ ignores the existence of eutectics, which are common for a glass-forming system and especially the existence of deep eutectics leads to discrepancies in the calculated critical cooling rates, which itself results in a shift of the experimentally observed minimum of the critical cooling rate. This is shown by the fact that the composition yielding the largest possible cast diameter for the bulk glasses presented in the work of Law et al. does not align with the composition yielding the minimum calculated critical cooling rate using the presented methods (see Figure S2) [25]. It should however be taken into account that the formation of deep eutectics is also the result of the same thermodynamic parameters, so that the determined composition for the minimum in Rc should still give a good description of the system, and just the numerical value of Rc might be lower in reality as estimated by the model. Secondly, the assumption that melts of vastly differing alloys that can be assumed to correspond to an averaged coordination number might by rather crude. Yet, if the mayor constituents stay within the range of transition metals, deviations might not be too large. Furthermore, the model in its current state is limited to 38 elements and in principle, since the enthalpies of mixing are based on the calculations done using the Miedema model, is limited to elements that are available by the cited Miedema calculator [11], which excludes elements like sulfur and oxygen. 

## 5. Conclusions

In this work a fast computational method was developed to calculate a set of thermodynamic parameters and the critical cooling rate for glass formation for all compositions of a chosen alloy system. Thus, a method is proposed to identify the subset of glass-forming compositions based on empirically found boundary conditions. The results are shown to coincide well with experimentally verified glass-forming compositions for Pd-Ni-P, Cu-Mg-Ca, Cu-Zr-Ti, and Ti-Zr-Cu-Ni.

The effects of micro-alloying with Co, Fe, Cu, and Gd on the glass-forming composition and their critical cooling rates are calculated for Pd-Ni-P-based alloys, showing (in agreement with experimental results) an insignificant influence on the obtained results for Co, Fe, and Cu and a significand influence for Gd.

An algorithm to procedurally generate potential glass-forming compositions with decreasing critical cooling rates for a given alloy system based on a random starting composition is presented and compared exemplarily to experimentally confirmed glass-forming compositions within the Ti-Zr-Cu-Ni system. Removing the need to calculate all possible compositions yields a more viable approach for alloys with a large number of components and provides a way to predict new glass-forming glass-forming alloys with a low critical cooling rate.

The script and a technical description of applications can be found in the Supplementary Materials.

## Supplementary Materials

The following are available online at https://www.mdpi.com/1099-4300/22/3/292/s1, Figure S1: Critical cooling rates and experimentally verified glasses for Cu-Mg-Ca (a) and Cu-Zr-Ti (b). Images correspond to Figure 5b,c, Figure S2: Critical cooling rates and experimentally verified glasses for Cu-Mg-Ca. Diameter of datapoints represents the diameter of cast glass samples ranging from 0.5 to 8 mm., Figure S3: Critical cooling rates of Zr-Ti-Cu-Ni for alloys found using the described “minimize-$R_{C}$”-method for a set 1000 found alloys (a). First 100 Datapoints of the same dataset (b). Corresponding to Figure 7c., Figure S4: Critical cooling rates of Zr-Ti-Cu-Ni for alloys found using the described “minimize-$R_{C}$”-method for a set 1000 found alloys sorted by descending values for $R_{C}$. Corresponding to Figure 7c. Table S1: Table of 50 compositions with low $R_{C}$ for 5 component systems found using the “minimize-$R_{C}$”-method utilizing 50 minimization steps. Table S2: Table of 50 compositions with low $R_{C}$ for 3 to 10 component systems found using the “minimize-$R_{C}$”-method utilizing 50 minimization steps., Source code and guide.
