# Supplementary material for: Exploring the Phase Space of Multi-Principal-Element Alloys and Predicting the Formation of Bulk Metallic Glasses
Source: Entropy (Basel). 2020 Mar 2;22(3):292. doi: 10.3390/e22030292 (PMC7516748; doi:10.3390/e22030292)
Supplement: Supplementary file 1 [file entropy-22-00292-s001.zip › Supplementary.docx]

| 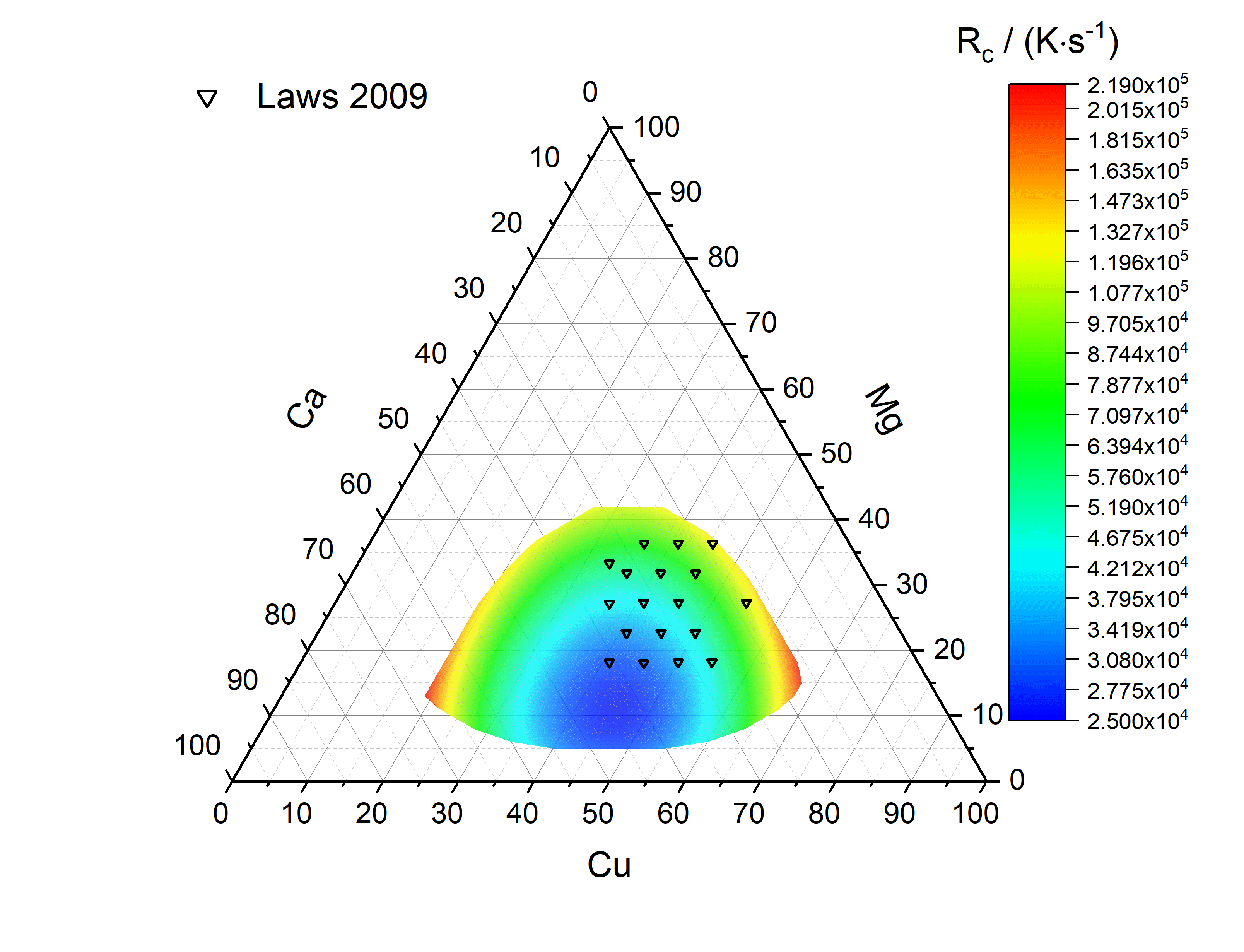  (**a**) | 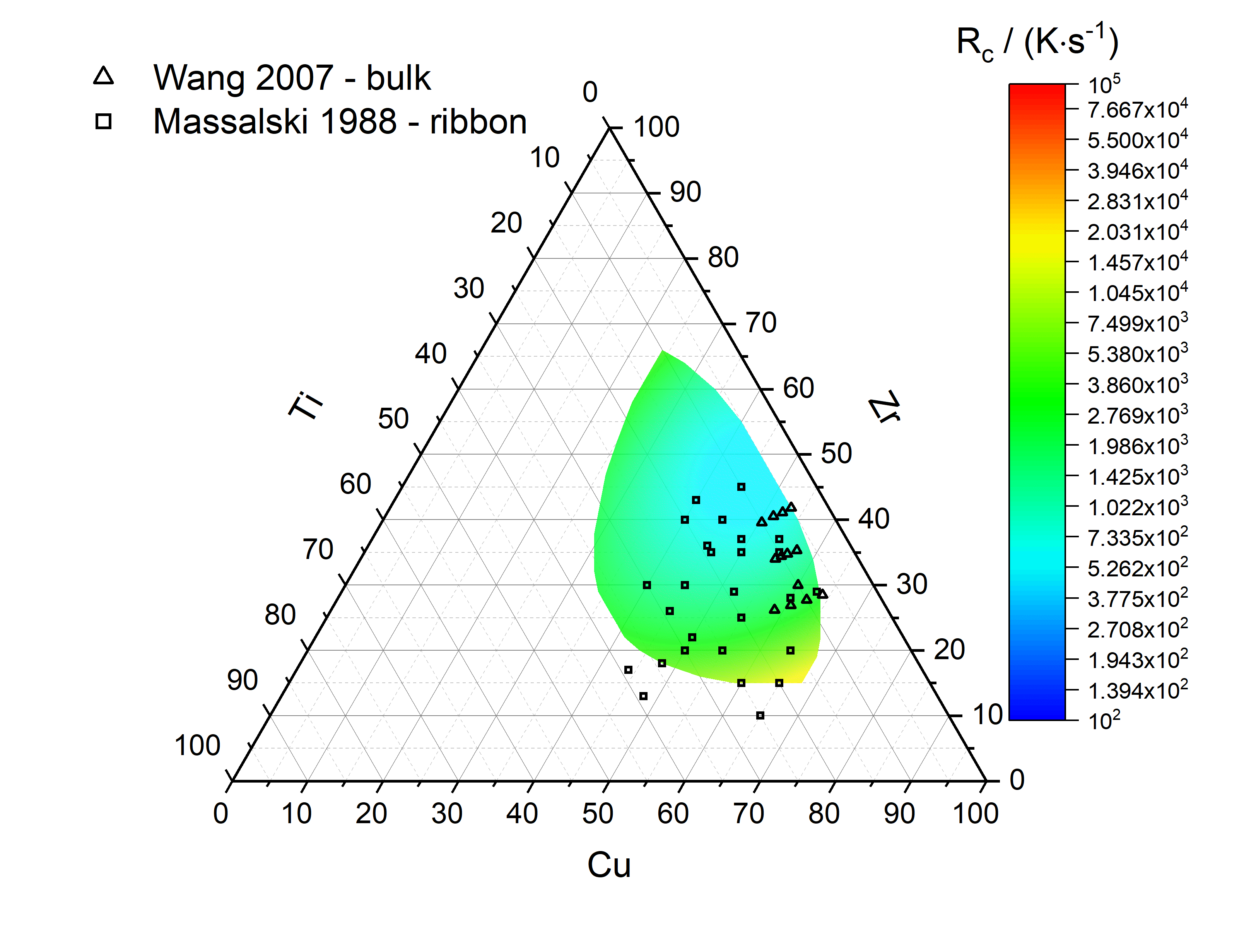  (**b**) |
| --- | --- |

**Figure S1:** Critical cooling rates and experimentally verified glasses for Cu-Mg-Ca (**a**) and Cu-Zr-Ti (**b**). Images correspond to Figure 5 (**b**) and (**c**).

| 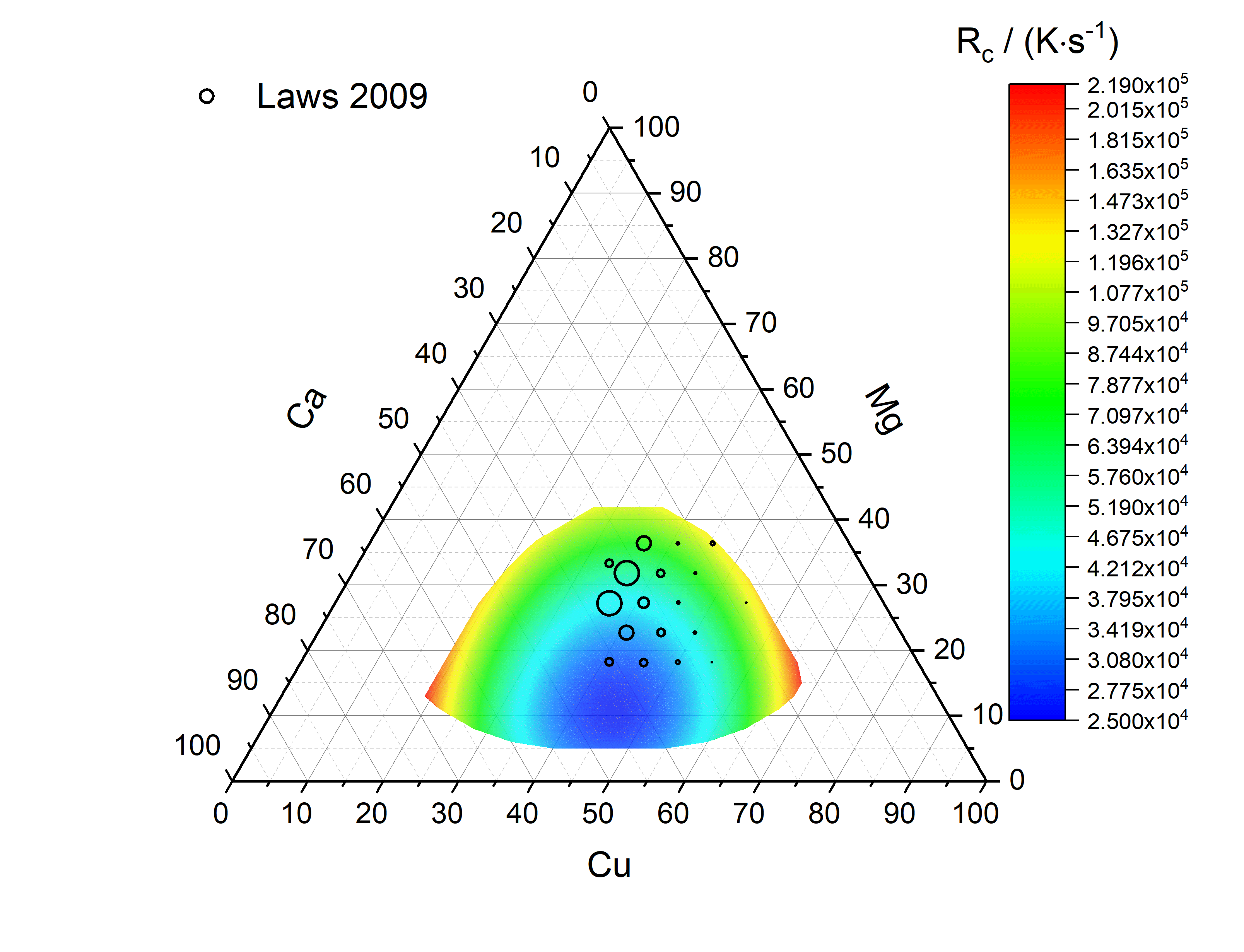 |
| --- |

**Figure S2:** Critical cooling rates and experimentally verified glasses for Cu-Mg-Ca. Diameter of datapoints represents the diameter of cast glass samples ranging from 0.5 to 8 mm.

| 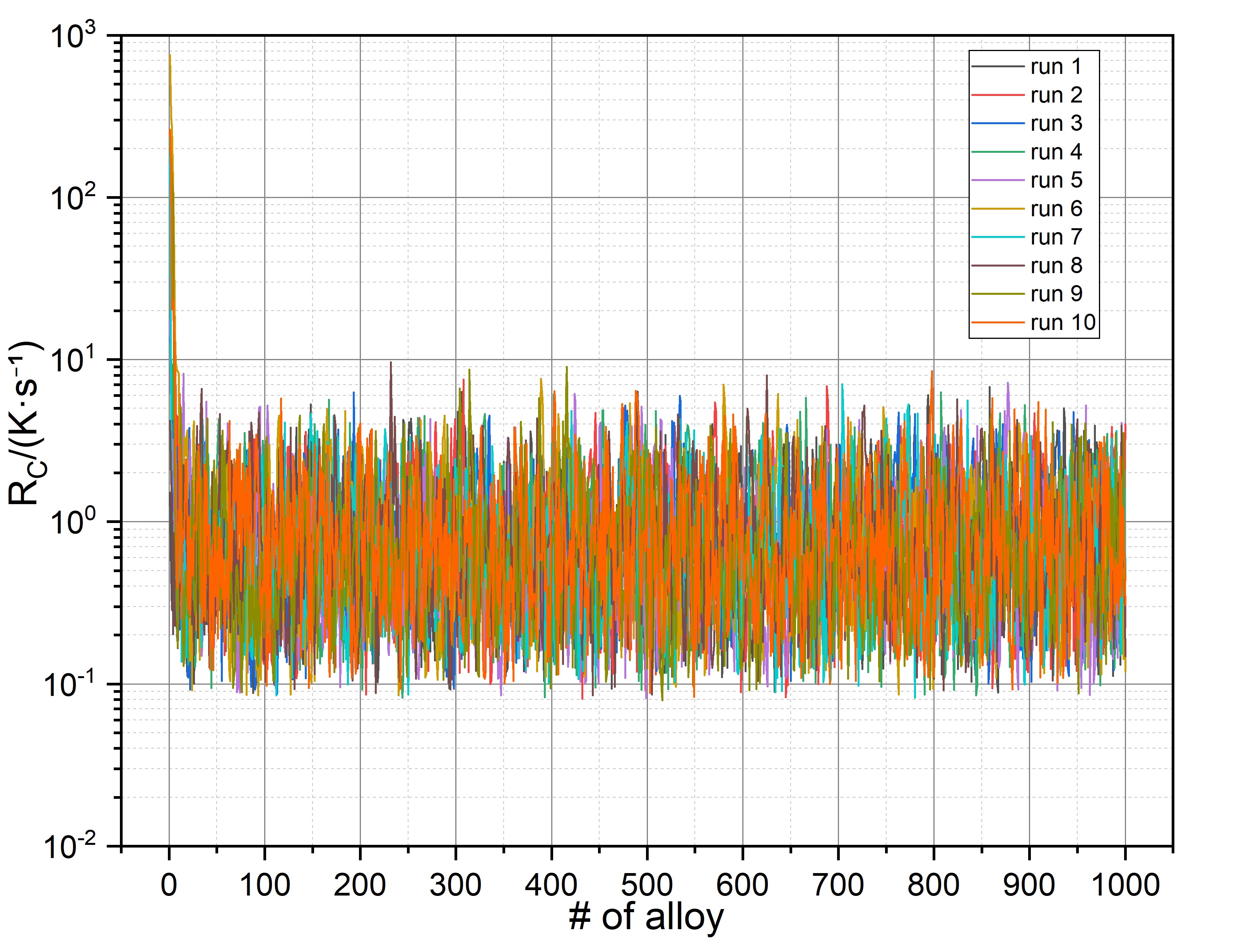  (**a**) | 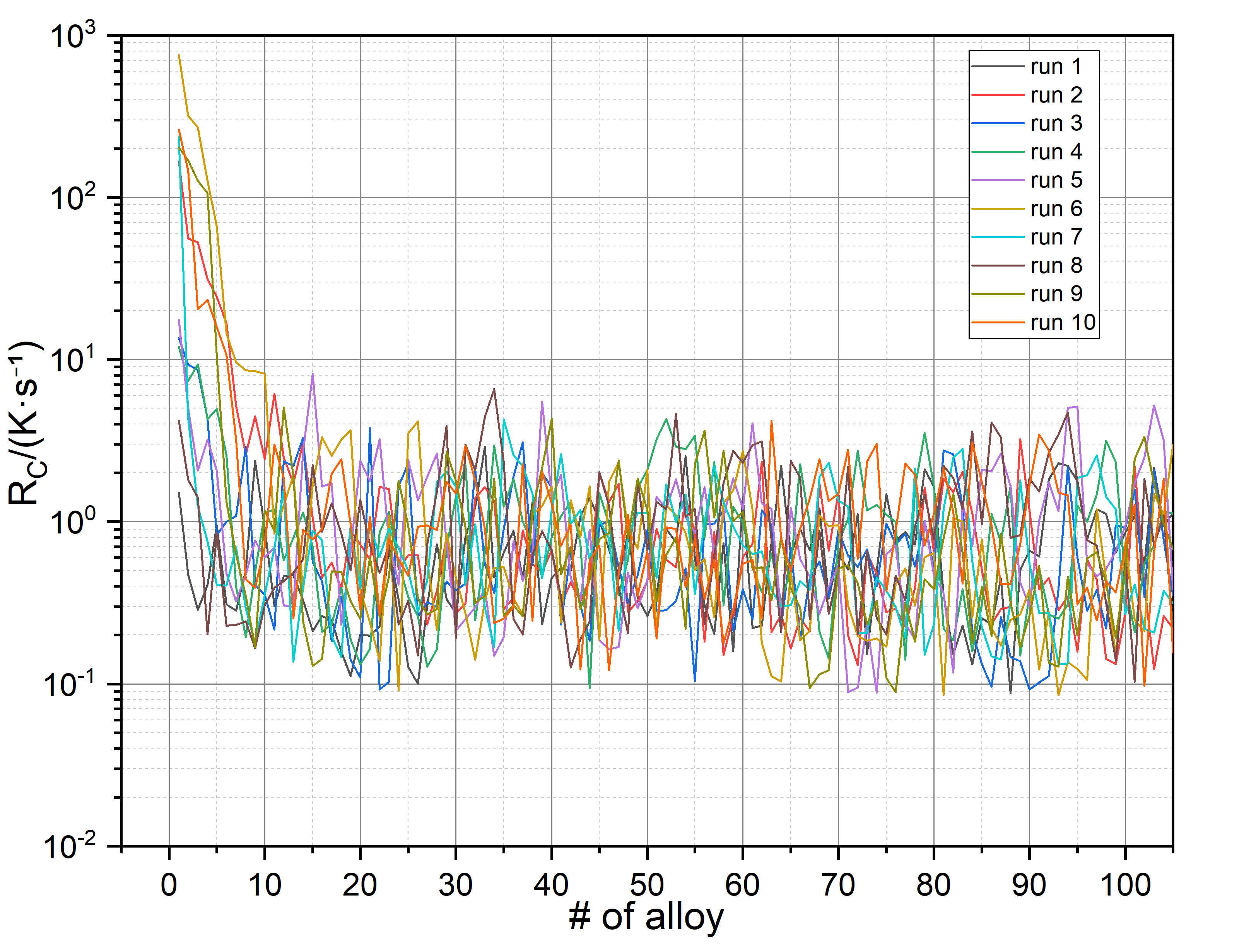  (**b**) |
| --- | --- |

**Figure S3:** Critical cooling rates of Zr-Ti-Cu-Ni for alloys found using the described “minimize-$R_{C}$”-method for a set 1000 found alloys (**a**). First 100 Datapoints of the same dataset (**b**). Corresponding to Figure 7 (**c**).

| 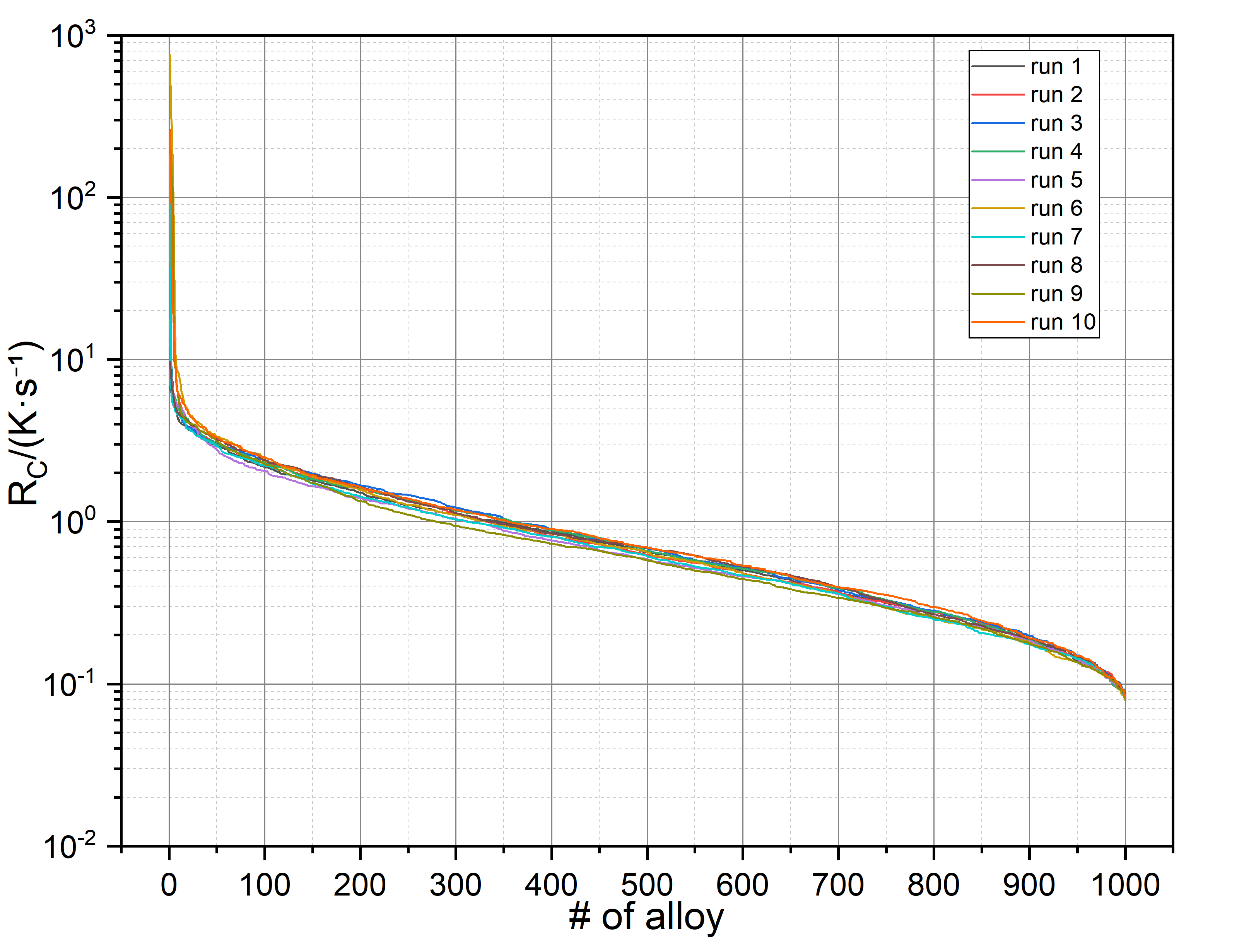 |
| --- |

**Figure S4:** Critical cooling rates of Zr-Ti-Cu-Ni for alloys found using the described “minimize-$R_{C}$”-method for a set 1000 found alloys sorted by descending values for $R_{C}$. Corresponding to Figure 7 (**c**).

**Table S1:** Table of 50 compositions with low $R_{C}$ for 5 component systems found using the “minimize-$R_{C}$”-method utilizing 50 minimization steps.

| **Alloy** | $\boldsymbol{R}_{\boldsymbol{C}}$**/(K/s)** | **Alloy** | $\boldsymbol{R}_{\boldsymbol{C}}$**/(K/s)** |
| --- | --- | --- | --- |
| Co₃₂Pd₂₉C₂₀Ag₁₂Sr₇ | 0.018 | Ti₁₇Hf₃₉Cu₁Be₄₀Tb₃ | 0.301 |
| Gd₂₄Ni₁₈Sn₁₂Si₂₂Li₂₄ | 0.297 | Ca₂₀Ag₂₂Tb₅₁Au₃P₄ | 0.414 |
| Ni₁₀Sn₂₇Nb₂₀Co₆Sc₃₇ | 0.111 | Al₁₉Gd₃₆Sn₁₈Sr₁₆Be₁₁ | 0.364 |
| Ni₃₁Sc₂₁Zn₁₀Ca₁₈Sn₂₀ | 0.208 | Ag₉W₁₂Ca₃₈Pd₃₄Li₇ | 0.104 |
| Ag₂₉Zn₉Hf₃₂Si₁₄Gd₁₆ | 0.068 | Pd₁₂Dy₁₅Nb₄₇Co₁₉Si₇ | 0.012 |
| C₃₂Cr₁₁Sn₂₁B₂₆Mn₁₀ | 0.009 | C₅Pd₆Li₅₅Si₁₅Dy₁₉ | 9.078 |
| Sn₅Ca₁₉Si₃₄Sr₈Hf₃₄ | 0.101 | Cr₁₆Mo₁₆Hf₂₀B₂₉Cu₁₉ | 0.004 |
| Gd₃₄Fe₁₅Sn₁₃Al₁₃Dy₂₅ | 0.103 | Mo₁₈Hf₃₆Cu₁Ni₂₇Sn₁₈ | 0.046 |
| V₁₁Co₂₂Yb₁₄Dy₃₀Sn₂₃ | 0.284 | Gd₁₂Y₉Al₂₆B₂₉Ge₂₄ | 0.193 |
| Fe₁₂Gd₃₁Co₂₀Al₃₃Sr₄ | 0.253 | C₁₈Si₁₂Cr₃₄Mg₁₃Au₂₃ | 0.013 |
| Fe₁₅C₇Nb₃₆Zr₁₄Ta₂₈ | 0.001 | Cr₅Y₂₆W₂₅Hf₁₇Pd₂₇ | 0.002 |
| Al₂₆P₁₁Ni₁₈Si₂₇Fe₁₈ | 0.310 | W₂₀Dy₁₈Hf₁₇Mo₈Ge₃₇ | 0.009 |
| Sr₂₅Pt₂₂B₁₈Cu₃₀Sc₅ | 0.065 | Nb₃₀Al₂₂Mo₁₈B₁₅Y₁₅ | 0.008 |
| Li₇Hf₃₈Si₂₃Mg₆Er₂₆ | 0.043 | Gd₃₆Mn₁₃Ge₁₈Ag₃₁Al₂ | 0.252 |
| Gd₁₃Cu₁₂Sc₃₇Nb₃Co₃₅ | 11.765 | Al₁₅Sn₂₈Sc₁C₄₇Ni₉ | 0.072 |
| Ni₃₁W₉Sc₃Zr₂₅Hf₃₂ | 0.011 | Cu₁₄P₁₀Gd₂₄Mo₂₆Au₂₆ | 0.034 |
| Sn₃₆Y₄₁Sr₉Ti₅Nb₉ | 0.339 | Sc₁₇Mo₁₉B₂₂Zn₁₂Au₃₀ | 0.020 |
| P₆Yb₂₂Mn₂₄Si₂₃Be₂₅ | 0.264 | Ge₃₇Sr₁Nb₁₈Sc₂₆W₁₈ | 0.059 |
| Al₃₁Yb₁₁Er₁₄Fe₁₂Hf₃₂ | 0.108 | Er₂₃W₂₈Cu₄C₁₂Sc₃₃ | 0.002 |
| Si₂₃Ti₃₂Ca₁₃Ni₂₀Ag₁₂ | 0.148 | Sr₄Co₂₃Al₅₀Mn₁₉Cu₄ | 42.617 |
| Tb₂₀Mn₂₀Be₁₂V₂₃B₂₅ | 0.022 | Nb₁₀C₉Pd₁₃W₃₄Tb₃₄ | 0.001 |
| Pd₁₈Sr₆Sc₁₅Mn₂₉Gd₃₂ | 0.059 | Cr₃₂Au₁₄Ag₁Ta₂₄Si₂₉ | 0.019 |
| V₂₁Ta₁₈Mn₂₈B₂₀Fe₁₃ | 0.010 | Si₂₆Al₁₅Yb₂₆Ca₁₇Sn₁₆ | 0.429 |
| C₄₆Al₁₂B₈Ag₂₇Si₇ | 0.171 | Sc₁₉Er₃₀Yb₁Ni₄₂Mn₈ | 0.769 |
| Y₂₄Dy₂₆Be₃₂Cu₁₆Fe₂ | 2.586 | Pt₂₆Mg₇Be₃₄B₁₈Yb₁₅ | 0.091 |

**Table S2:** Table of 50 compositions with low $R_{C}$ for 3 to 10 component systems found using the “minimize-$R_{C}$”-method utilizing 50 minimization steps.

| **Alloy** | $\boldsymbol{R}_{\boldsymbol{C}}$**/(K/s)** | **Alloy** | $\boldsymbol{R}_{\boldsymbol{C}}$**/(K/s)** |
| --- | --- | --- | --- |
| Y₈Ti₅Co₃₃Er₁₆Hf₂₉Si₇Ca₁Al₁ | 0.007 | Fe₄₃Be₁₉Al₃₈ | 5153.194 |
| Y₄₁Pd₉Yb₂₇Be₂₃ | 0.295 | Gd₁P₁Mn₃₂Sc₁Al₁₁Li₁Ca₅Ni₃₁Sn₁₆Sr₁ | 13.795 |
| Dy₄₀Zn₁₆B₁₂Zr₃₂ | 0.153 | Gd₃₀Tb₂₂P₁Zn₁B₁₆Cu₁₂Hf₁₈ | 0.012 |
| Zr₁C₈Y₄Pd₁₁Cr₃₈Be₁₂Ge₂₄Li₂ | 0.012 | Cu₉Nb₂₂P₁₂Mg₈Mn₃₁Sc₁₈ | 0.049 |
| Dy₂₇P₅Ag₄Ni₁Sc₅₄Ge₁Cr₅Ca₁Mg₁Pd₁ | 1.029 | Tb₂₄Y₁Hf₈Zn₁Ti₅₀Li₃P₅Zr₂B₅Yb₁ | 7.474 |
| Dy₂₈Co₁Ag₄₄Tb₂₇ | 9.145 | Fe₁Er₁₁Zr₃₅Ca₆Pt₃Ti₁₀Si₄Co₃₀ | 0.010 |
| Co₆Tb₇Sc₅₀Pt₉Mg₁Mo₁₂Sn₁Au₆Er₂Nb₆ | 0.014 | Si₆Cr₁₃Sr₁Ge₁P₁₂Zr₁Ti₁Pd₁Ni₁₂Ca₅₂ | 3.629 |
| Sc₁₀Nb₁Al₂Ni₅₂Cu₂Y₉Dy₂₁B₁Hf₁Pt₁ | 0.052 | Co₃₉Mg₃V₂Li₂Si₂₄Zn₁₇Hf₁₂Al₁ | 0.073 |
| Be₁Hf₁Gd₁V₁Sn₂₈Ag₁Co₈Er₂₇Cr₂₉Nb₃ | 10.350 | Be₁Pd₁₀Zr₁₄Yb₁Mg₁₂Gd₄₅C₁Nb₁Si₃Li₁₂ | 0.412 |
| Pd₆Ge₁₅Co₃Ca₈B₁Zn₆Hf₄₃Mo₁₈ | 0.004 | Co₂₉Dy₇V₂₈Pt₃₆ | 0.066 |
| Ta₁Ca₁Tb₁Si₁Mo₁₂Sn₁Pt₇₀Au₁B₁₂ | 0.374 | Sc₈Cu₅₁Ca₂Yb₃₁P₃Ta₁Fe₁Li₁B₂ | 110.204 |
| Zn₁₈Yb₁Al₁₇Sr₃Dy₈Gd₃₅Y₁₈ | 0.097 | Y₄₃Zn₄₈Fe₉ | 19.067 |
| Hf₃₄Ni₁₇Ti₂₂Dy₁₅Pd₄Li₁Ca₁Si₆ | 0.003 | Sn₆C₂₃Fe₃₀Dy₅Be₂₄Sr₁₂ | 0.011 |
| Er₁Sn₂₁Y₃Mg₇Cu₃₁Yb₂Dy₂₅Zr₁₀ | 0.203 | Al₂₁Gd₃Ag₉Be₂₇Pd₃₂Ca₅P₃ | 0.112 |
| Ag₇Sn₂₁V₃₇Al₅P₁₉Zn₁₁ | 0.505 | Y₁W₁Sc₄Gd₂Yb₁Ta₂₀Mo₇Zn₅₈Tb₅Sr₁ | 3009.811 |
| V₁Be₃₇Cu₁₄Y₄₈ | 18.775 | Ni₄₉Fe₂Yb₄Gd₄₅ | 24.624 |
| Co₃Er₁₁Gd₂Ag₁Ni₂₅Be₂Si₃₂W₂₄ | 0.005 | Pt₁₉Mg₁₄Sr₂₈Sc₂₃Ca₁₅Sn₁ | 0.058 |
| C₂₁Si₂₉Zr₂B₁₅Li₁₄Au₁₈Hf₁ | 0.009 | Sn₃₇Fe₂Sr₃Ni₃₈Cr₂Nb₃Pd₁Tb₂Zn₉Zr₃ | 34.324 |
| Ta₂₅Hf₃₁Cr₁₈Ag₂Ge₂₄ | 0.010 | V₁₄Gd₂₄Ag₆₂ | 71583.174 |
| C₅₁Zn₃₇Sn₁₂ | 8.737 | Si₄Li₃₁Ag₃₄P₃₁ | 6.073 |
| Ca₃₀Ag₁₆V₂₀Pd₃₄ | 0.150 | Mn₂₄Sn₆₆Fe₁₀ | 86267.939 |
| Zn₄₈Tb₄₇Hf₅ | 4.547 | Nb₃₆Fe₂₈Pd₃₆ | 0.117 |
| Si₁₇Al₂₃C₃₉Au₂₁ | 0.078 | Mn₉Yb₂₀Al₃₆Y₃₅ | 0.630 |
| Fe₁Sc₉B₄₅Hf₁C₁Mg₂₆Zn₁Dy₁Tb₃Sr₁₂ | 33.240 | Sc₂₃Ca₈C₁₁Dy₃Gd₃₆Ti₁₉ | 0.012 |
| Y₄Mn₁Hf₂₈Au₆Ta₂₂Ge₂₀W₁₉ | 0.001 | V₁Tb₉Ta₂₅Y₅Co₂₈Mo₄Cu₂Be₂₆ | 1.040 |
