# Supplementary material for: Exploring the Phase Space of Multi-Principal-Element Alloys and Predicting the Formation of Bulk Metallic Glasses
Source: Entropy (Basel). 2020 Mar 2;22(3):292. doi: 10.3390/e22030292 (PMC7516748; doi:10.3390/e22030292)
Supplement: Supplementary file 1 [file entropy-22-00292-s001.zip › ParaCal-QuickGuide.pdf]

# ParaCal – Quick Guide (QM)

In the following we provide a quick guide to the use of the *python* script that was used to implement the presented computations.

## 1. Basic information and usage

The shown examples are all executed using *IPython* and *Jupyter Notebook* for ease of use and visualization.

The `.ipynb` and `.py` files should be arrange using the following folder structure:

..\YourFolderName\ParaCal Ver.5.ipynb

..\YourFolderName \alloyGenerator.py

..\YourFolderName \paraCalLib.py

..\YourFolderName \database.py

..\YourFolderName \database\atomic_weights.txt

..\YourFolderName \database\atomicRadii.txt

..\YourFolderName \database\densities.txt

..\YourFolderName \database\electronegativity.txt

..\YourFolderName \database\elements.txt

..\YourFolderName \database\meltingTemperatures.txt

..\YourFolderName \database\mixing_enthalpies_AB.txt

..\YourFolderName \database\valenceElectronConc.txt

Open `ParaCal Ver.5.ipynb` using *Jupyter Notebook* and execute the first cell to import the function libraries and load the database by clicking into it and pressing CTRL + Enter, see Figure QG 1 below.

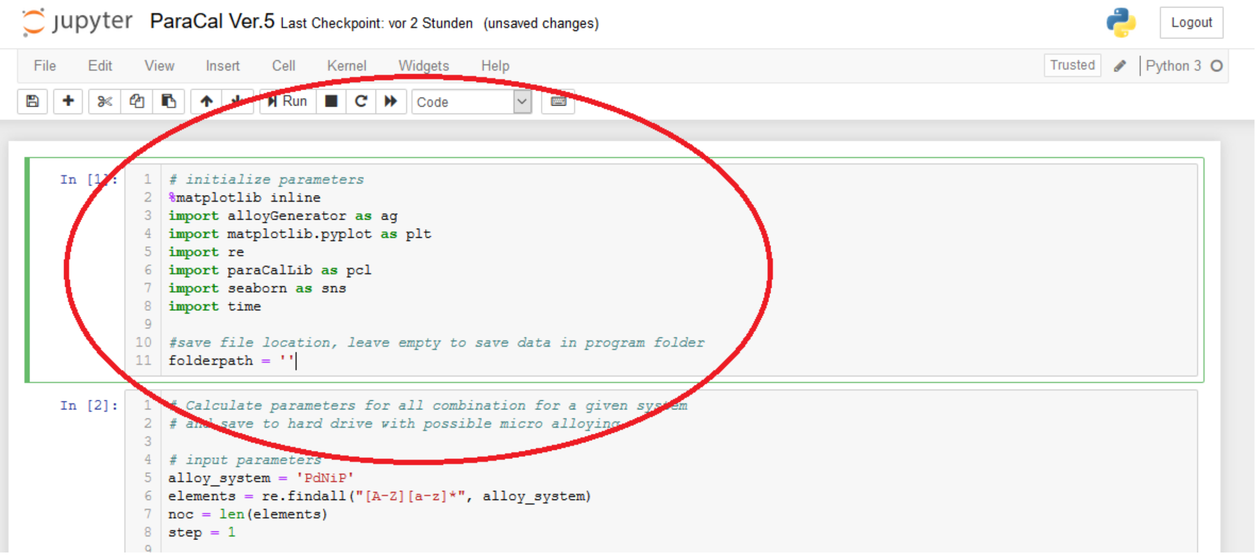


Figure QG1: Initialization parameters and initialization steps of the ParaCal python script.

Furthermore, the user can use the variable **folderpath** to specifiy the saving location of calculated data.

# 2. Calculations of alloy systems: Glass-Formation ability and Micro-Alloying

The calculation of a complete alloy system (using the method described in Figure 1 of the manuscript) is carried out using the code in the second cell:

1. Define the alloy system using the **alloy_system** variable

2. Set the at.% step-size used in the calculation using the **step** variable

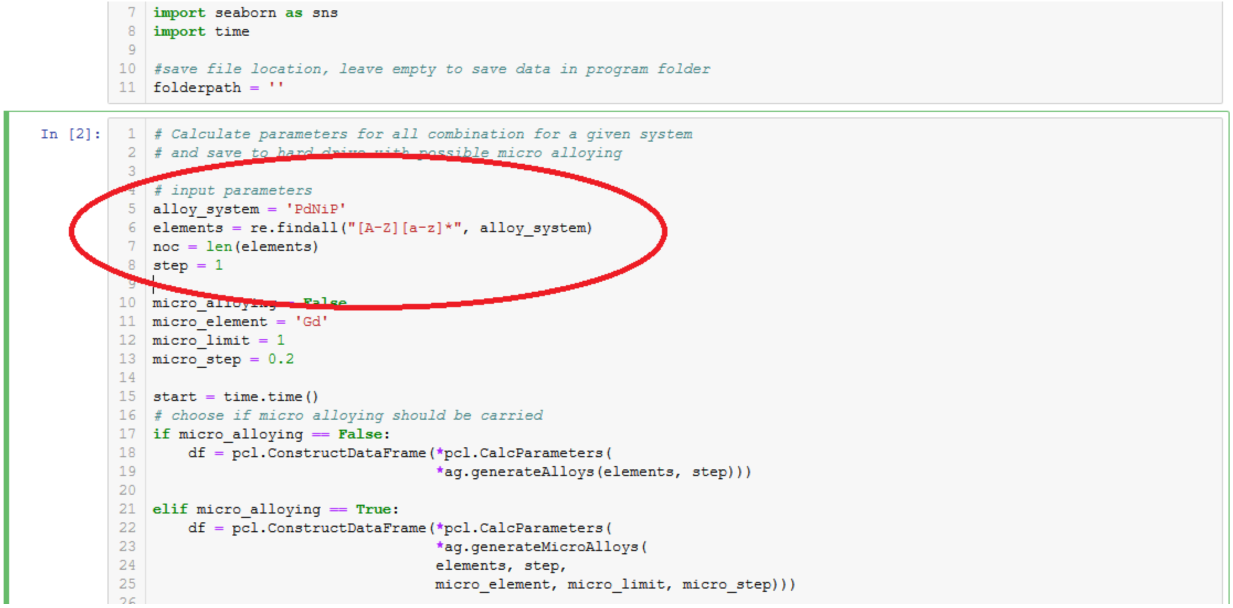


Figure QG2: Definition of the alloy system. The elements for the alloy are defined using the **alloy_system** variable. The number of components or elements, **noc,** and the number of output glass-candidates, **nog**. Step defines the atomic concentration **step**s.

3. For micro-alloying set **micro_alloying = True**, choose the element to by used, the maximum percentage for the micro-alloyed element and the step-size for different micro-alloying concentrations using **mirco_element**, **micro_limit** and **micro-step**, respectively.

```python
import seaborn as sns
import time

#save file location, leave empty to save data in program folder
folderpath = ''
```

In [2]:

```python
# Calculate parameters for all combination for a given system
# and save to hard drive with possible micro alloying

# input parameters
alloy_system = 'PdNiP'
elements = re.findall("[A-Z][a-z]*", alloy_system)
noc = len(elements)
step = 1

micro_alloying = False
micro_element = 'Gd'
micro_limit = 1
micro_step = 0.2

start = time.time()
# choose if micro alloying should be carried
if micro_alloying == False:
    df = pcl.ConstructDataFrame(*pcl.CalcParameters(
                                *ag.generateAlloys(elements, step)))

elif micro_alloying == True:
    df = pcl.ConstructDataFrame(*pcl.CalcParameters(
                                *ag.generateMicroAlloys(
                                elements, step,
                                micro_element, micro_limit, micro_step)))
```

Figure QG3: Add elements for a micro-alloying study. **micro_alloying** = True sets the analysis to an micro_element with a limit of the maximum concentration, **micro_limit**, and a concentration step up to this maximum concentration, **micro_step**.

4. Execute the cell by pressing CTRL + Enter.

This will yield a `.csv` – file in the chosen folder or in the folder that contains `ParaCal Ver.5.ipynb` if **folderpath** is left empty. The file will be named as `“alloy_system”.csv` without as `“alloy_system+micro_element”_microalloyed.csv`.

# 3. Search for New Glasses

Cell Number 3 contains an example of the **findBMGs()**-function which is used to generate the starting BMG-candidate used in the described “find-and-vary”- and “minimize-$R_C$”-methods.

**findBMGs()** takes two parameters: The number of components or elements **noc** and the number of glass-candidates it should generate **nog**. Executing cell 3 using with **noc=3** and **nog=10** yield a list of 10 glass-candidate with 3 randomly chosen elements each:

```
In [3]:
#find nog random bulk metallic glasses with noc components
noc = 3
nog = 10
pcl.findBMGs(noc, nog)
```

Out[3]:

| | ΔHmix/(kJ·mol⁻¹) | ΔSmix/(J·K⁻¹·mol⁻¹) | δ/(%) | R_C/(K·s⁻¹) | is BMG? |
|---|---|---|---|---|---|
| $Cr_{46}Yb_{36}Pd_{18}$ | -9.071971 | 8.594336 | 14.264160 | 26018.067905 | True |
| $Ca_{55}Pt_{7}Mg_{38}$ | -16.284784 | 7.338679 | 11.699071 | 9157.365337 | True |
| $Ge_{20}Be_{60}Nd_{20}$ | -21.299200 | 7.900986 | 15.836526 | 1237.373215 | True |
| $Be_{39}Nb_{36}Er_{25}$ | -17.909424 | 8.992881 | 17.696024 | 265.888284 | True |
| $B_{25}V_{31}Cu_{44}$ | -16.822265 | 8.903720 | 17.507331 | 1095.298674 | True |
| $Er_{19}Sr_{13}Zn_{68}$ | -21.348920 | 7.009240 | 17.102982 | 1631.993826 | True |
| $Ni_{43}Nb_{33}Be_{24}$ | -27.422180 | 8.907062 | 9.045323 | 6.862795 | True |
| $Cu_{9}Ni_{52}Gd_{39}$ | -26.746075 | 7.682429 | 18.326569 | 38.823316 | True |
| $C_{64}B_{18}Co_{18}$ | -31.988851 | 7.507551 | 20.811463 | 0.820208 | True |
| $Pd_{37}B_{50}Cu_{13}$ | -29.610283 | 8.145474 | 24.573280 | 8.667130 | True |

Figure QG4: Result of a general search for a glass with a certain number of components or elements, **noc**, and a number of output glasses, **nog**.

It is possible to impose constraints in the form desired elements with or without desired atomic percentages on the **findBMGs()** function. For example handing over a list of list of two elements and a list of their desired atomic percentages (here **['Zr', 'Y']** and **[40, 10]**) yields, for **noc=4** and **nog=10**, a list of 10 4-component glass candidates, where just 2 elements and their atomic percentages are chosen by the function:

```
In [8]:
#find nog random bulk metallic glasses with noc components and given elements and composition
noc = 4
nog = 10
elements_given = ['Zr', 'Y']
comp_given = [40, 10]
pcl.findBMGs(noc, nog, elements_given, comp_given)
```

Out[8]:

| | ΔHmix/(kJ·mol⁻¹) | ΔSmix/(J·K⁻¹·mol⁻¹) | δ/(%) | R_C/(K·s⁻¹) | is BMG? |
|---|---|---|---|---|---|
| $Zr_{40}Y_{10}Cr_{38}Ni_{12}$ | -19.141896 | 10.134398 | 14.190897 | 46.471868 | True |
| $Zr_{40}Y_{10}Fe_{34}Ni_{16}$ | -26.179947 | 10.449473 | 14.408546 | 3.275371 | True |
| $Zr_{40}Y_{10}Ni_{31}Sr_{19}$ | -15.460555 | 10.604100 | 19.615944 | 311.925862 | True |
| $Zr_{40}Y_{10}V_{43}Au_{7}$ | -10.519055 | 9.526959 | 11.247540 | 2417.262377 | True |
| $Zr_{40}Y_{10}Al_{16}Cr_{34}$ | -27.854440 | 10.449473 | 12.665385 | 1.741719 | True |
| $Zr_{40}Y_{10}Pt_{2}Cu_{48}$ | -23.854994 | 8.541615 | 12.950276 | 59.117000 | True |
| $Zr_{40}Y_{10}Fe_{48}Ta_{2}$ | -17.454718 | 8.541615 | 14.295315 | 455.319949 | True |
| $Zr_{40}Y_{10}Be_{17}Cu_{33}$ | -26.940029 | 10.508364 | 15.413432 | 4.649496 | True |
| $Zr_{40}Y_{10}Nd_{26}Ni_{24}$ | -22.340944 | 10.721678 | 11.519284 | 18.000042 | True |
| $Zr_{40}Y_{10}Cr_{33}Fe_{17}$ | -13.138339 | 10.508364 | 14.268371 | 408.256815 | True |

Figure QG5: Result of a general search for a glass with a certain number of components or elements, **noc**, and a number of output glasses, **nog**. The **elements_given** constrains elements with **comp_given** atomic percentages (here **['Zr', 'Y']** and **[40, 10]**).

To use the described “find-and-vary”-method one needs to call the **find_and_vary_BMG()**-function, which itself uses the **findBMGs()**-function to find a starting glass-candidate. **find_and_vary_BMG()** additionally takes a parameter called **number_of_tries** to specify a breaking condition for the function. This means the function will stop its search after **number_of_tries** variations of the compositions did not

yield a new glass-candidate. The example shows this for 10 candidates within the PdNiP-System:

In [9]:

```python
# find a metallic glass and vary its composition to find related glasses
elem_given = ['Pd', 'Ni', 'P']
comp_given = []
noc = 3
nog = 10
number_of_tries = 10000

df = pcl.find_and_vary_BMG(noc, nog, number_of_tries , elem_given, comp_given)

element_columns = [e.strip() for e in re.findall('[A-Z][a-z]?[ ]', " ".join(list(df.columns)))]
atprc_columns = [e + '/(at%)' for e in element_columns]
columns = dict(zip(element_columns, atprc_columns))
df.rename(columns = columns, inplace = True)

df
```

```
Number of tries exceeded maximum number of tries.
```

Out[9]:

| | Pd/(at%) | Ni/(at%) | P/(at%) | $\Delta$Hmix/(kJ·mol$^{-1}$) | $\Delta$Smix/(J·K$^{-1}$·mol$^{-1}$) | $\delta$/(%) | R_C/(K·s$^{-1}$) | is BMG? |
|---|---|---|---|---|---|---|---|---|
| **$Pd_{57}Ni_{26}P_{17}$** | 57 | 26 | 17 | -27.747992 | 8.080654 | 9.058315 | 32.813609 | True |
| **$Pd_{50}Ni_{30}P_{20}$** | 50 | 30 | 20 | -31.324040 | 8.561009 | 9.455174 | 6.754339 | True |
| **$Pd_{51}Ni_{30}P_{19}$** | 51 | 30 | 19 | -30.141829 | 8.481884 | 9.313492 | 10.913510 | True |
| **$Pd_{58}Ni_{25}P_{17}$** | 58 | 25 | 17 | -27.770619 | 8.013044 | 9.068054 | 33.706827 | True |
| **$Pd_{57}Ni_{24}P_{19}$** | 57 | 24 | 19 | -30.294884 | 8.135322 | 9.409123 | 12.201308 | True |
| **$Pd_{55}Ni_{25}P_{20}$** | 55 | 25 | 20 | -31.458730 | 8.291774 | 9.548691 | 7.287191 | True |
| **$Pd_{59}Ni_{23}P_{18}$** | 59 | 23 | 18 | -29.088040 | 7.965190 | 9.256483 | 21.011455 | True |
| **$Pd_{63}Ni_{17}P_{20}$** | 63 | 17 | 20 | -31.671613 | 7.601101 | 9.638371 | 9.689628 | True |
| **$Pd_{53}Ni_{25}P_{22}$** | 53 | 25 | 22 | -33.716763 | 8.448881 | 9.832284 | 2.913918 | True |
| **$Pd_{49}Ni_{30}P_{21}$** | 49 | 30 | 21 | -32.466109 | 8.634311 | 9.589673 | 4.268055 | True |

Figure QG6: Result of find and vary approach.

The next cell depicts a variant of the “minimize-$R_C$”-method that strictly minimizes the critical cooling rate:

In [36]:

```
#strictly minize R_C starting from a given or random BMG
elem_given = ['Zr','Ti', 'Cu', 'Ni']
comp_given = []
noc = 5
nog = 10
number_of_tries = 10000

df = pcl.minimize_rc(noc, nog, number_of_tries, elem_given, comp_given)

# graph if R_C vs alloy number
alloy_number = [i for i in range(len(df.index))]

sns.lineplot(x = alloy_number,
             y = df['R_C/(K·s⁻¹)'])
plt.yscale('log')
plt.show()

element_columns = [e.strip() for e in re.findall('[A-Z][a-z]?[ ]', " ".join(list(df.columns)))]
atprc_columns = [e + '/(at%)' for e in element_columns]
columns = dict(zip(element_columns, atprc_columns))
df.rename(columns = columns, inplace = True)

df
```

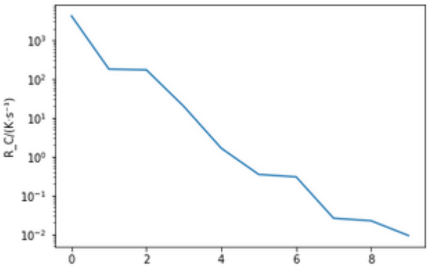


Out[36]:

| | Zr/(at%) | Ti/(at%) | Cu/(at%) | Ni/(at%) | C/(at%) | ΔHmix/(kJ·mol⁻¹) | ΔSmix/(J·K⁻¹·mol⁻¹) | δ/(%) | R_C/(K·s⁻¹) | is BMG? |
|---|---|---|---|---|---|---|---|---|---|---|
| $Zr_2Ti_{48}Cu_{47}Ni_1C_2$ | 2 | 48 | 47 | 1 | 2 | -15.575052 | 7.563651 | 9.385650 | 4224.364965 | True |
| $Zr_4Ti_{44}Cu_{47}Ni_1C_4$ | 4 | 44 | 47 | 1 | 4 | -21.585358 | 8.477871 | 11.448808 | 179.001847 | True |
| $Zr_3Ti_{41}Cu_{42}Ni_{12}C_2$ | 3 | 41 | 42 | 12 | 2 | -19.756737 | 9.709412 | 9.719143 | 171.891421 | True |
| $Zr_2Ti_{39}Cu_{42}Ni_{13}C_4$ | 2 | 39 | 42 | 13 | 4 | -24.405434 | 10.008961 | 11.323319 | 19.592975 | True |
| $Zr_2Ti_{39}Cu_{40}Ni_{13}C_6$ | 2 | 39 | 40 | 13 | 6 | -29.551928 | 10.359962 | 12.846226 | 1.635174 | True |
| $Zr_2Ti_{40}Cu_{41}Ni_9C_8$ | 2 | 40 | 41 | 9 | 8 | -33.354888 | 10.219181 | 14.186714 | 0.345246 | True |
| $Zr_7Ti_{38}Cu_{33}Ni_{17}C_5$ | 7 | 38 | 33 | 17 | 5 | -31.568691 | 11.396697 | 12.783588 | 0.298272 | True |
| $Zr_3Ti_{42}Cu_{31}Ni_{16}C_8$ | 3 | 42 | 31 | 16 | 8 | -37.804888 | 11.040647 | 14.425393 | 0.025590 | False |
| $Zr_{13}Ti_{28}Cu_{47}Ni_2C_{10}$ | 13 | 28 | 47 | 2 | 10 | -38.937675 | 10.684236 | 16.401444 | 0.022064 | False |
| $Zr_{14}Ti_{27}Cu_{45}Ni_4C_{10}$ | 14 | 27 | 45 | 4 | 10 | -40.037185 | 11.200557 | 16.508849 | 0.009164 | False |

Figure QG7: Result to strictly minimize $R_c$, the minimize the cooling rate.

The fore last cell in the `ParaCal Ver.5.ipynb` contains an example of the more flexible "minimize-$R_C$"-method presented in the manuscript. The examples show this done for a list of 10 minimization steps for a 4-component system:

```
In [10]:
# minize R_C with chance of accepting higher R_C
# elem_given = ['Zr','Ti', 'Cu', 'Ni']
elem_given = []
comp_given = []
noc = 4
nog = 10
number_of_tries = 10000

df = pcl.minimize_rc_basic_MonteCarlo(noc, nog, number_of_tries, elem_given, comp_given)
#df.to_csv(folderpath + '/' + 'ParacalExplorerTest.csv', sep = ';', encoding="utf-8-sig")
#df.sort_values(by=['r_c/(K/s)'], ascending = False)
alloy_number = [i + 1  for i in range(len(df.index))]

sns.lineplot(x = alloy_number,
             y = df['R_C/(K·s⁻¹)'])
plt.yscale('log')
plt.title('R_C/(K·s⁻¹) vs alloy number')
plt.show()

desc_rc = df.sort_values(by=['R_C/(K·s⁻¹)'], ascending = False)

sns.lineplot(x = alloy_number,
             y = desc_rc['R_C/(K·s⁻¹)'])
plt.yscale('log')
plt.title('R_C/(K·s⁻¹) vs alloy number; sorted for descending R_C')
plt.show()

sns.scatterplot(x = df['ΔHmix/(kJ·mol⁻¹)'], y = df['R_C/(K·s⁻¹)'])
plt.title('ΔHmix/(kJ·mol⁻¹) vs R_C/(K·s⁻¹)')
plt.show()

sns.scatterplot(x = df['ΔSmix/(J·K⁻¹·mol⁻¹)'], y = df['R_C/(K·s⁻¹)'])
plt.title('ΔSmix/(J·K⁻¹·mol⁻¹) vs R_C/(K·s⁻¹)')
plt.show()

sns.scatterplot(x = df['δ/(%)'], y = df['R_C/(K·s⁻¹)'])
plt.title('δ/(%) vs R_C/(K·s⁻¹)')
plt.show()

element_columns = [e.strip() for e in re.findall('[A-Z][a-z]?[ ]', " ".join(list(df.columns)))]
atprc_columns = [e + '/(at%)' for e in element_columns]
columns = dict(zip(element_columns, atprc_columns))
df.rename(columns = columns, inplace = True)

for e in atprc_columns:
    sns.scatterplot(x = df[e],
                    y = df['R_C/(K·s⁻¹)'])
    plt.yscale('log')
    plt.title('R_C/(K·s⁻¹) vs ' + e)
    plt.show()

df[df['is BMG?'] == True].sort_values(by=['R_C/(K·s⁻¹)'], ascending = True)
```

Figure QG8: Example of 10 minimization steps for a 4-component system.

And produces a list of the generate glasses sorted by ascending critical cooling rate:

```
df[df['is BMG?'] == True].sort_values(by=['R_C/(K·s⁻¹)'], ascending = True)
```

Out[10]:

| | Ag/(at%) | Fe/(at%) | Nd/(at%) | B/(at%) | ΔHmix/(kJ·mol⁻¹) | ΔSmix/(J·K⁻¹·mol⁻¹) | δ/(%) | R_C/(K·s⁻¹) | is BMG? |
|---|---|---|---|---|---|---|---|---|---|
| $Ag_2Fe_{44}Nd_{19}B_{35}$ | 2 | 44 | 19 | 35 | -34.878196 | 9.332554 | 25.358919 | 0.511866 | True |
| $Ag_{20}Fe_{14}Nd_{31}B_{35}$ | 20 | 14 | 31 | 35 | -32.635677 | 11.038675 | 27.466444 | 0.516188 | True |
| $Ag_2Fe_{46}Nd_{17}B_{35}$ | 2 | 46 | 17 | 35 | -34.308280 | 9.180112 | 24.901392 | 0.713843 | True |
| $Ag_{22}Fe_{14}Nd_{32}B_{32}$ | 22 | 14 | 32 | 32 | -31.398379 | 11.121437 | 26.561870 | 0.853119 | True |
| $Ag_{32}Fe_1Nd_{32}B_{35}$ | 32 | 1 | 32 | 35 | -33.827074 | 9.501161 | 27.393089 | 1.073185 | True |
| $Ag_4Fe_{42}Nd_9B_{45}$ | 4 | 42 | 9 | 45 | -33.346560 | 8.889395 | 24.931910 | 1.274201 | True |
| $Ag_6Fe_{42}Nd_{21}B_{31}$ | 6 | 42 | 21 | 31 | -30.216989 | 10.176547 | 24.821350 | 1.940112 | True |
| $Ag_{34}Fe_5Nd_{29}B_{32}$ | 34 | 5 | 29 | 32 | -29.572015 | 10.311464 | 26.203437 | 3.542613 | True |
| $Ag_{43}Fe_1Nd_{33}B_{23}$ | 43 | 1 | 33 | 23 | -30.654023 | 9.252688 | 22.743557 | 5.291583 | True |
| $Ag_{46}Fe_1Nd_{35}B_{18}$ | 46 | 1 | 35 | 18 | -30.273582 | 8.974265 | 20.459738 | 7.703950 | True |

Figure QG9: Example output list.

Where the compositions with the lowest calculated critical cooling rate would be the preferred glass forming candidates.

The last cell in `ParaCal Ver.5.ipynb` shows an example how calling this function multiple time using a loop and just picking the candidate with the lowest critical cooling rate for each iteration of the loop can be used to generate a list of different promising candidates:

```
In [5]:
# minize R_C with chance of accepting higher R_C
import pandas as pd
import random
pd.set_option('display.max_rows', 10000)
elem_given = ['Fe']
comp_given = []
nog = 10
number_of_tries = nog*nog

minimalRCList = []

for run in range(1,51):
    noc = random.randint(3,10)
    df = pcl.minimize_rc_basic_MonteCarlo(noc, nog, number_of_tries, elem_given, comp_given)


    element_columns = [e.strip() for e in re.findall('[A-Z][a-z]?[ ]', " ".join(list(df.columns)))]
    atprc_columns = [e + '/(at%)' for e in element_columns]
    columns = dict(zip(element_columns, atprc_columns))
    df.rename(columns = columns, inplace = True)

    minimalRCList.append([df.loc[df['R_C/(K·s⁻¹)'].idxmin()].name,
                          df.loc[df['R_C/(K·s⁻¹)'].idxmin()]['R_C/(K·s⁻¹)']])


print()
print('Alloy, r_c')
for e in minimalRCList:
    print(e[0],e[1])
```

```
Alloy, r_c
Fe19Au3Zr51Pt4Cu5Y6Yb2Mo9Ca1 0.6235511739553136
Fe4Si2Au8Ta1Nb20Co1Yb27Be35Sr1Ag1 52.113620424233964
Fe1Zn52Dy44Nb2Pd1 4.392353151369605
Fe11Tb29Cu1Yb29C10Dy20 0.03927884688583749
Fe56Si5Cu2Mn1Ag1Sc15Gd8Tb1Pt10Nd1 7.406962616662618
Fe12V2Cr32Zr1Y27Au20Gd2Si2Be2 0.6784515271778631
Fe33Cr13Sn16C22Gd4Ag12 0.025717308406046625
Fe14B34Au13Pt22W11Si5Tb1 0.006893414304204546
Fe6Ag50Sc16Yb19Si9 33.60804468961607
Fe38Pt22Yb40 117.95938685800196
Fe21P37Sc2Y1Ag1Ge5B33 1.2747213376913717
Fe56Hf13Mn2Sn8Li1Ge2Pd3Cr1W7B7 8.905736165815433
Fe12Mo16Yb31Mg1Ge19Li1Er1Zr19 46.28086903193897
Fe3Mn26Al29Gd16Dy26 0.3825933855658306
Fe23Zn30Zr47 2.4743879143692142
Fe1Sn8Be40Hf29W19Co3 1.2108798919543688
```

Figure QG10: Example multiple usage to generate a list of different promising candidates.

This is method used to generate the alloy list displayed in the Tables S1 and S2 in the supplementary material.

# 4. Adding elements, extending the database

Additional elements can be added by the user by adding them and the appropriate numerical values into the corresponding `.txt` files (in the database folder), while keeping the alphabetical order and units intact. The scheme for the element pairs added to the `mixing_enthalpies_AB.txt` file is as follows:

For the example of a list four elements A, B, C and D the following pairs exist:

A-B

A-C

A-D

B-C

B-D

C-D
