# Supplementary figures and images for: Exploring the Phase Space of Multi-Principal-Element Alloys and Predicting the Formation of Bulk Metallic Glasses
Source: Entropy (Basel). 2020 Mar 2;22(3):292. doi: 10.3390/e22030292 (PMC7516748; doi:10.3390/e22030292)

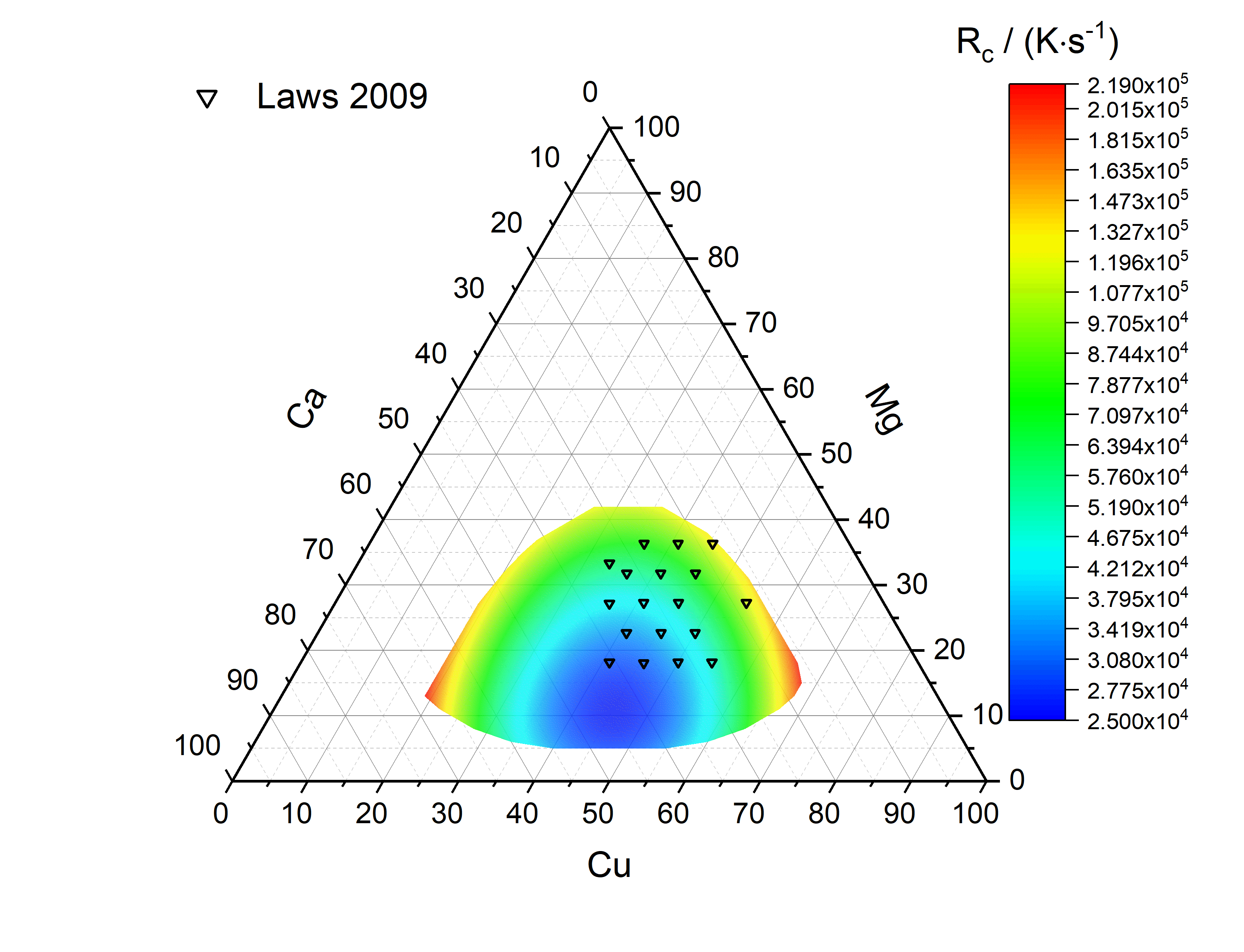

Supplement: Supplementary file 1 [file entropy-22-00292-s001.zip › CuMgCa_RC.png]

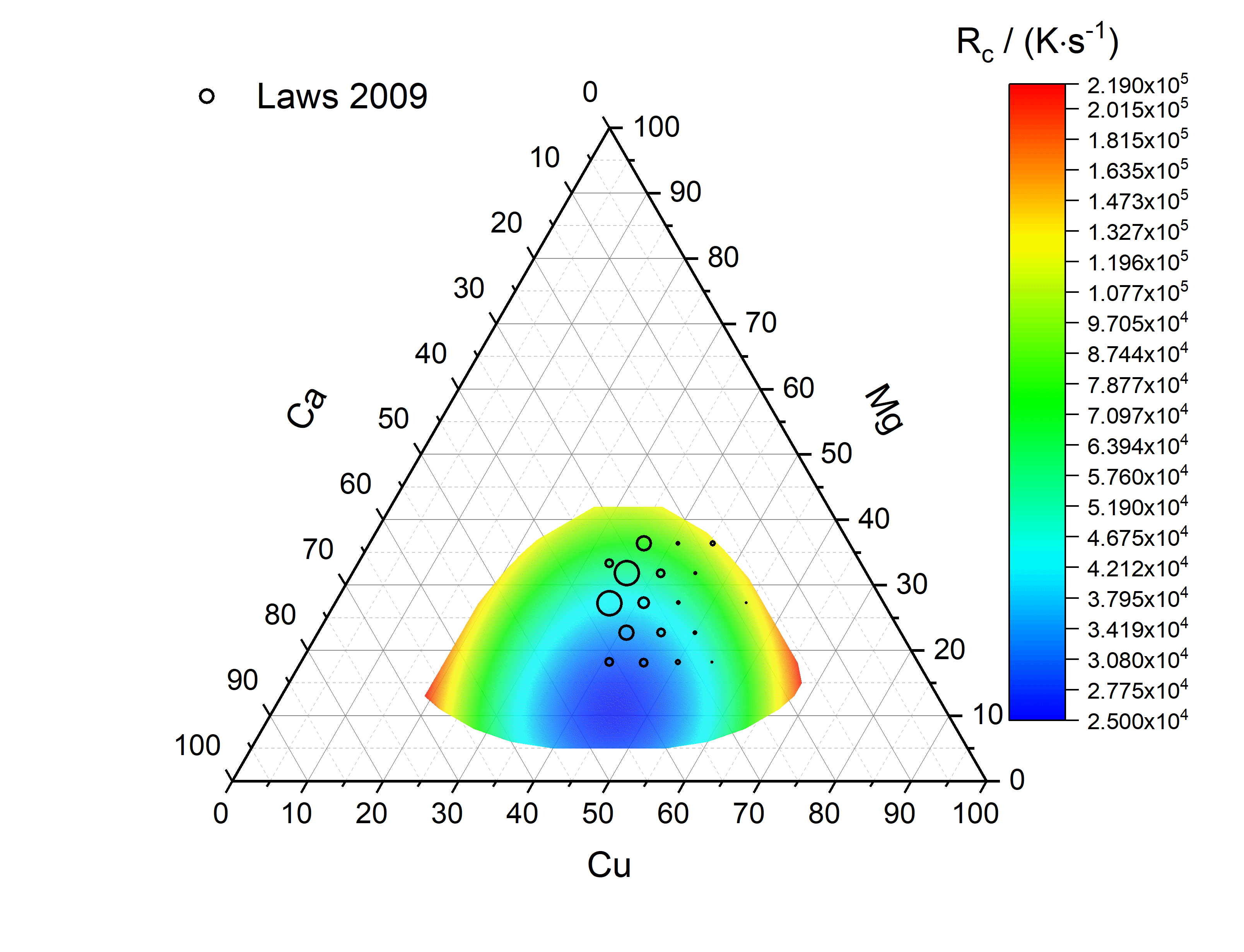

Supplement: Supplementary file 1 [file entropy-22-00292-s001.zip › CuMgCa_RC_vs_castDiameter.png]

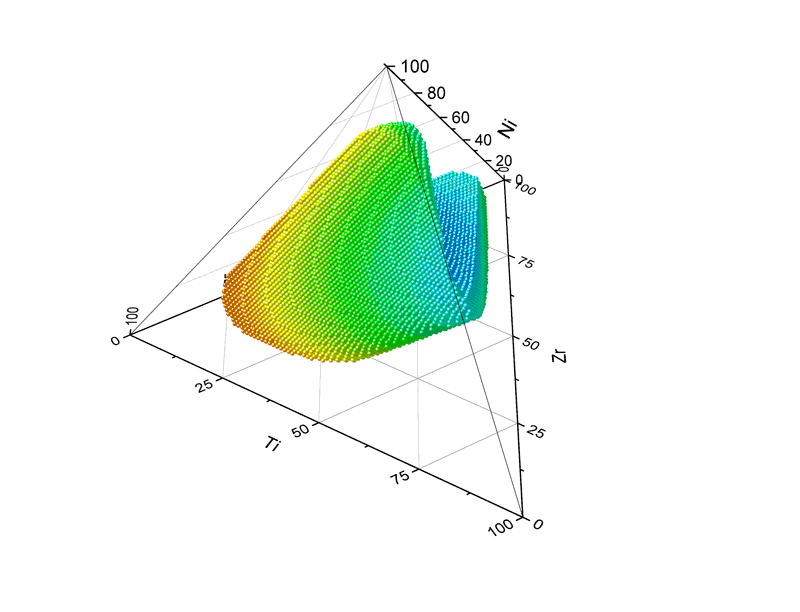

Supplement: Supplementary file 1 [file entropy-22-00292-s001.zip › CuZrTi_animation.gif]

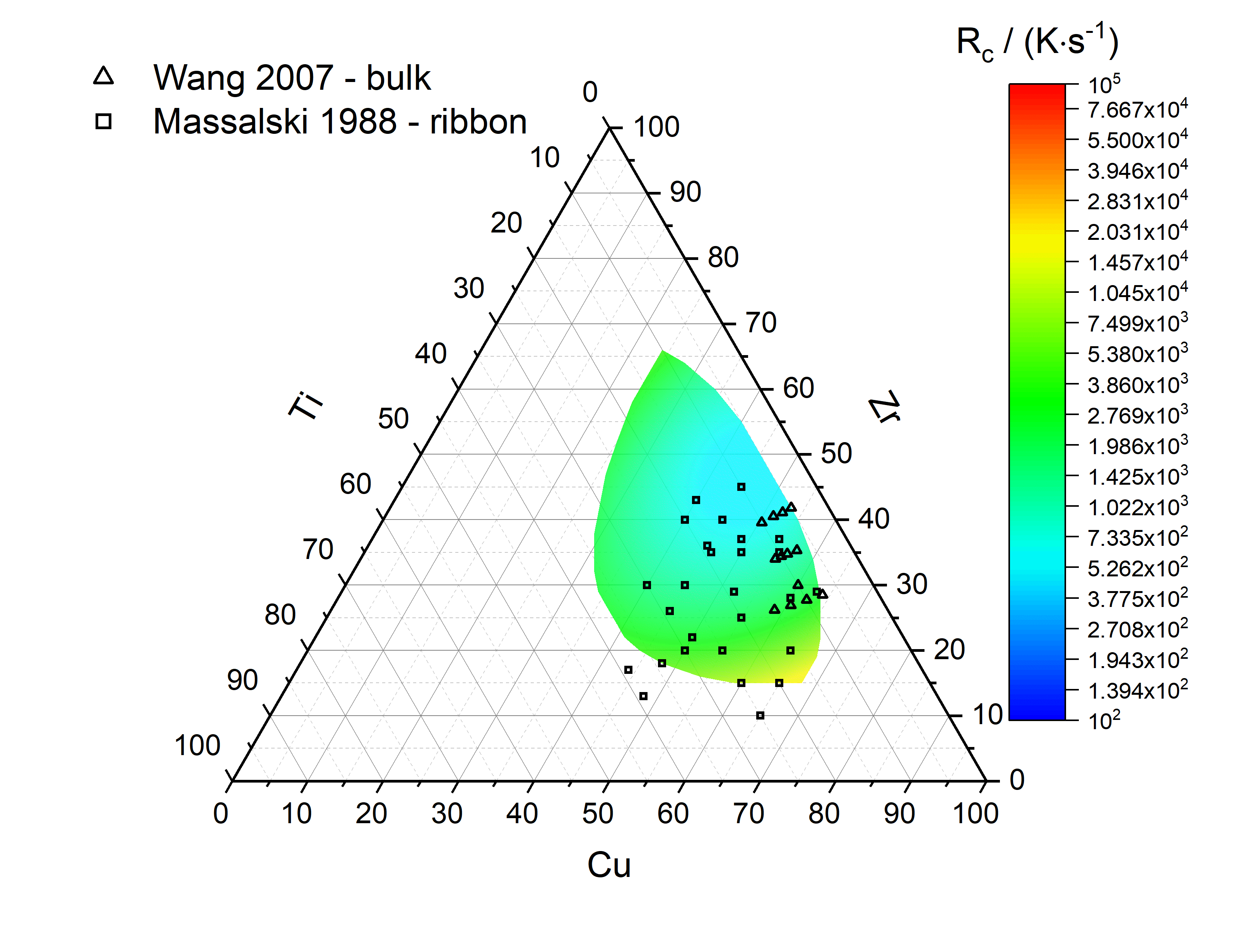

Supplement: Supplementary file 1 [file entropy-22-00292-s001.zip › CuZrTi_RC.png]

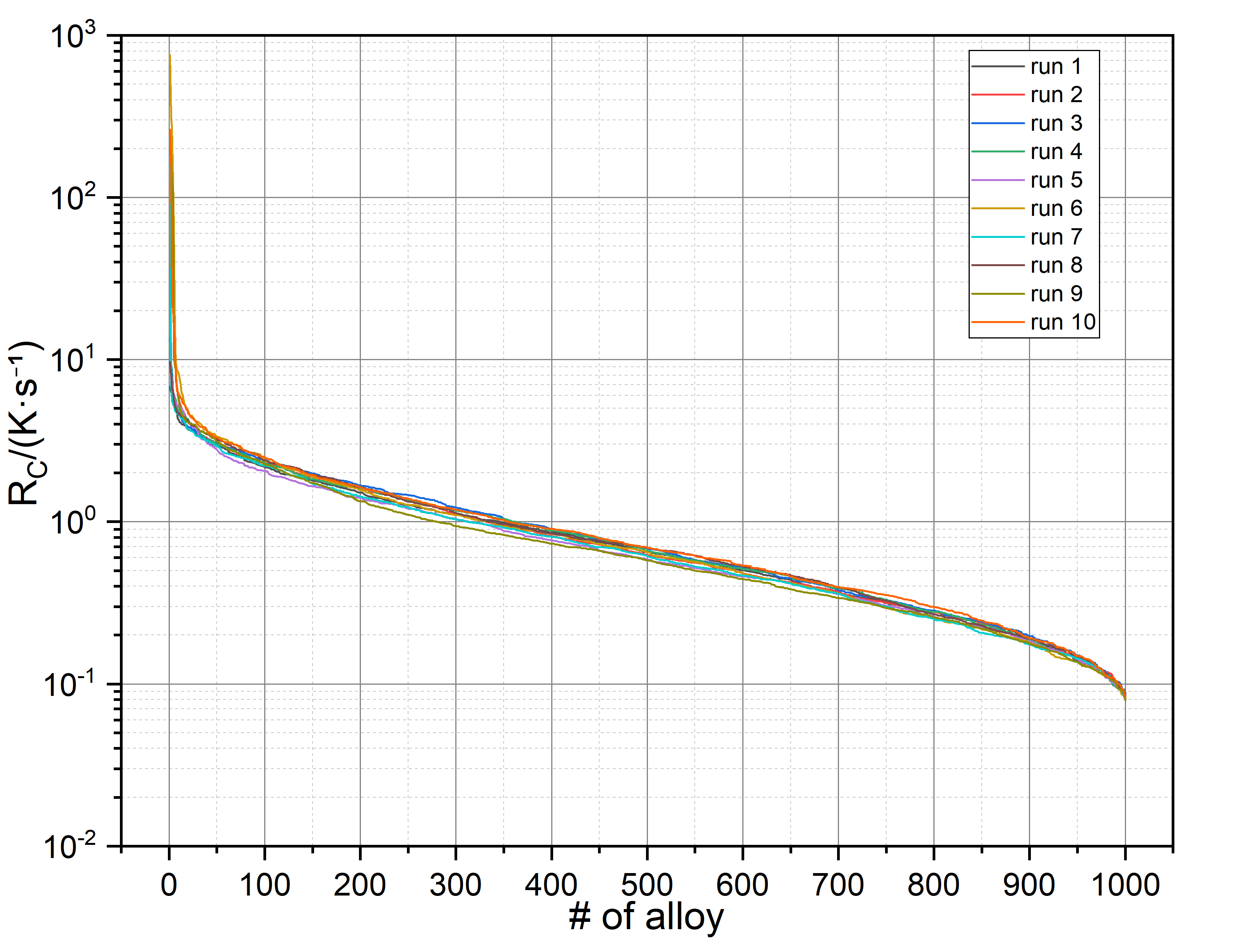

Supplement: Supplementary file 1 [file entropy-22-00292-s001.zip › ZrTiCuNi R_c sorted.png]

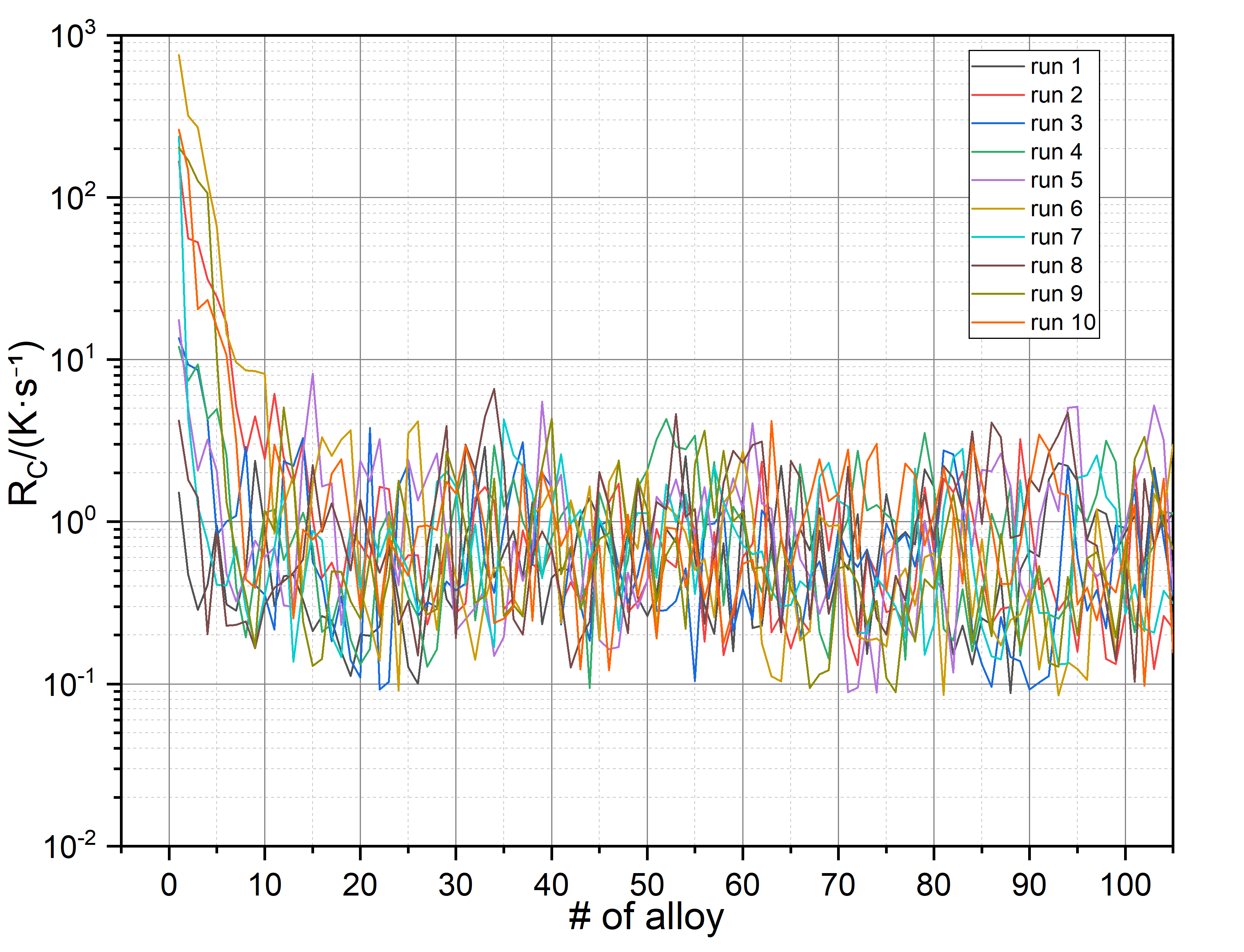

Supplement: Supplementary file 1 [file entropy-22-00292-s001.zip › ZrTiCuNi R_c unsorted zoomed.png]

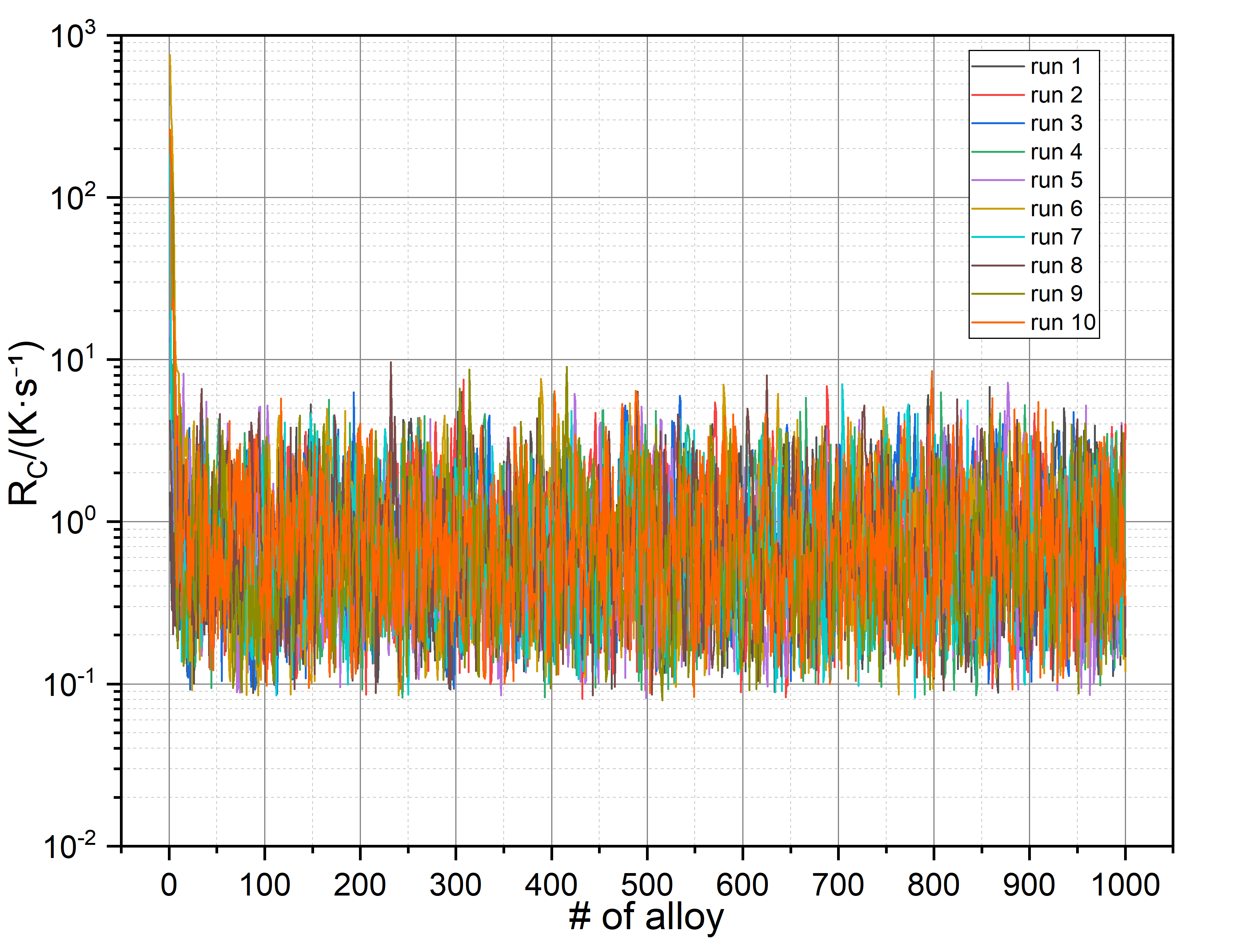

Supplement: Supplementary file 1 [file entropy-22-00292-s001.zip › ZrTiCuNi R_c unsorted.png]

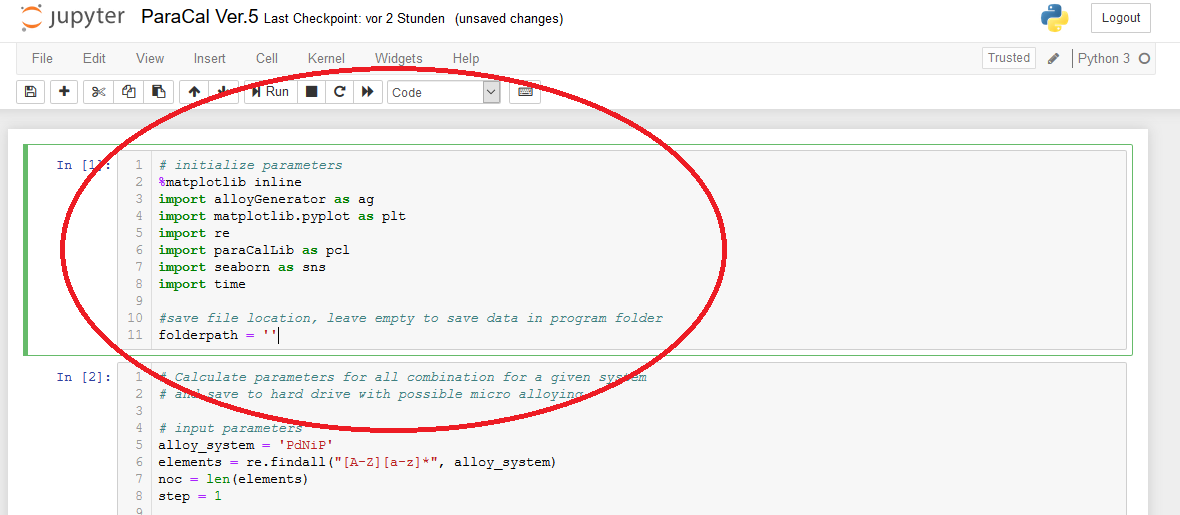

Supplement: Supplementary file 1 [file entropy-22-00292-s001.zip › QuickGuide/Step1.png]

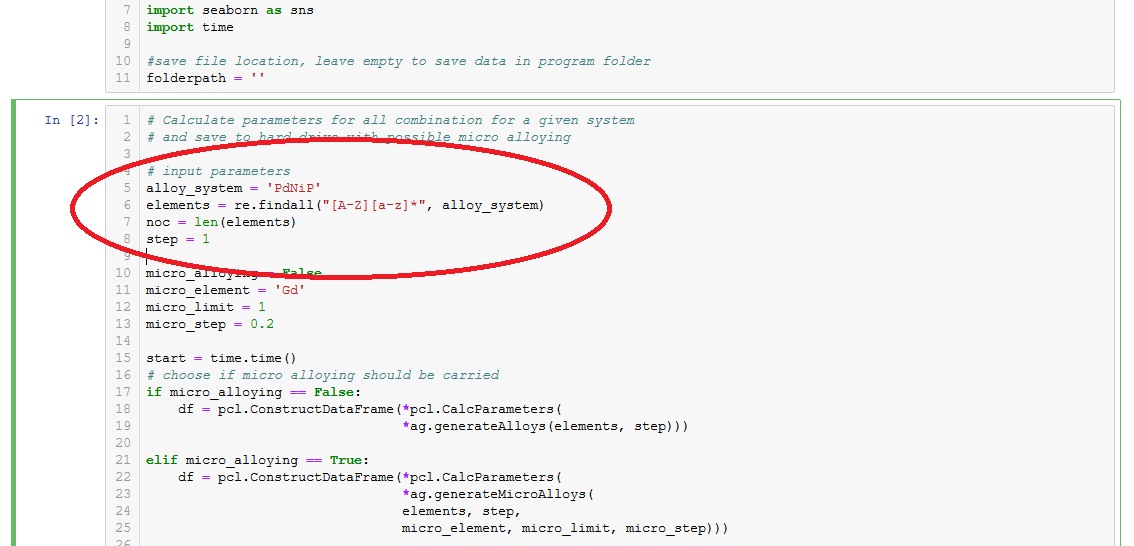

Supplement: Supplementary file 1 [file entropy-22-00292-s001.zip › QuickGuide/Step2a.png]

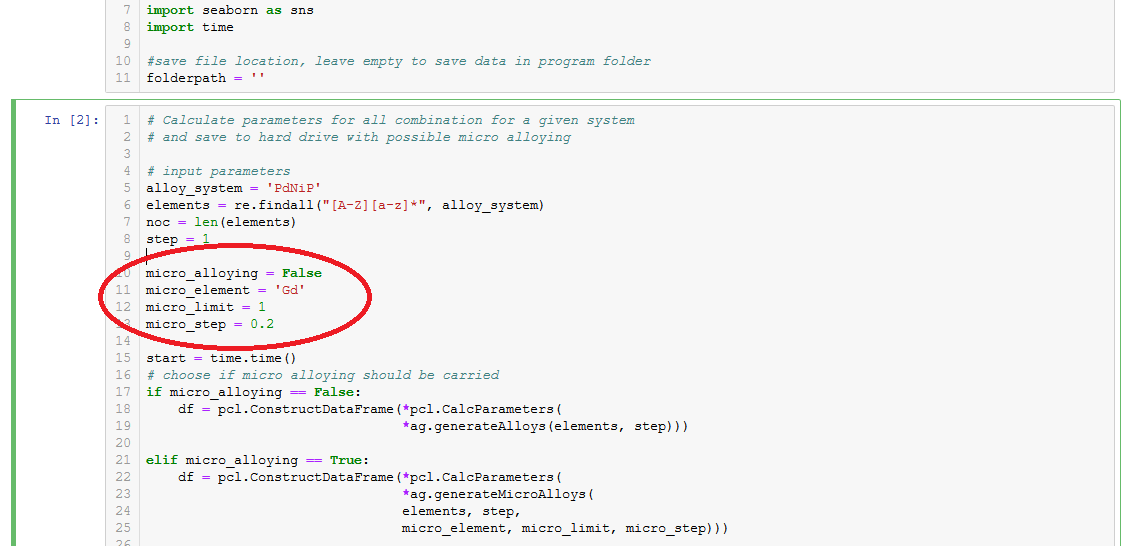

Supplement: Supplementary file 1 [file entropy-22-00292-s001.zip › QuickGuide/Step2b.png]

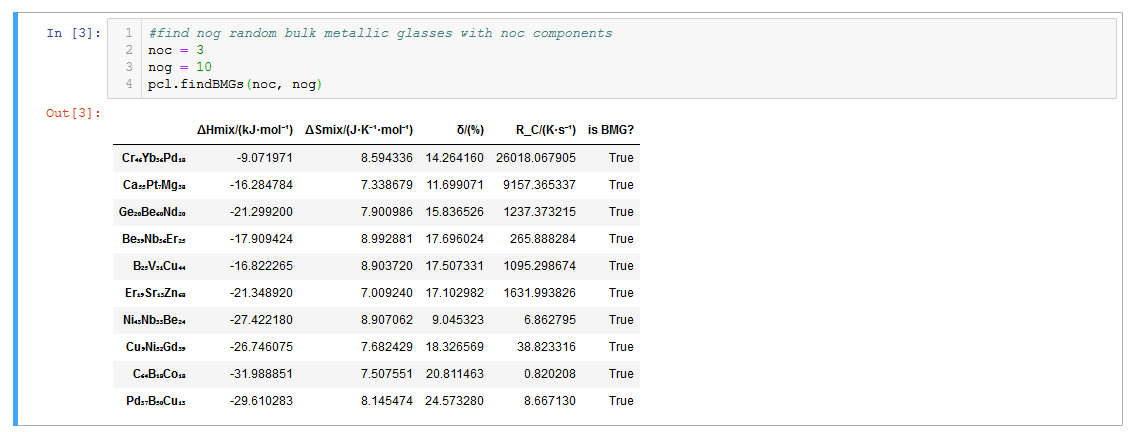

Supplement: Supplementary file 1 [file entropy-22-00292-s001.zip › QuickGuide/Step3.png]

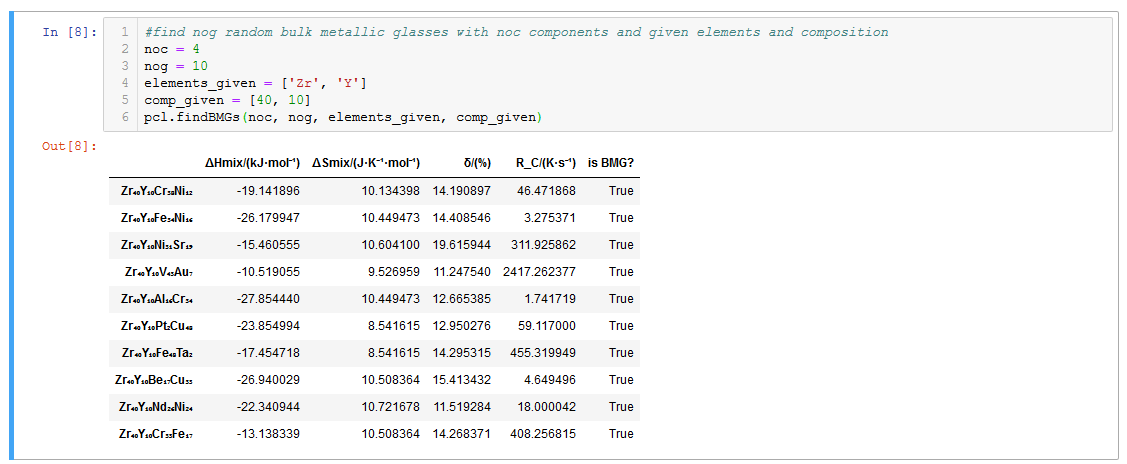

Supplement: Supplementary file 1 [file entropy-22-00292-s001.zip › QuickGuide/Step4.png]

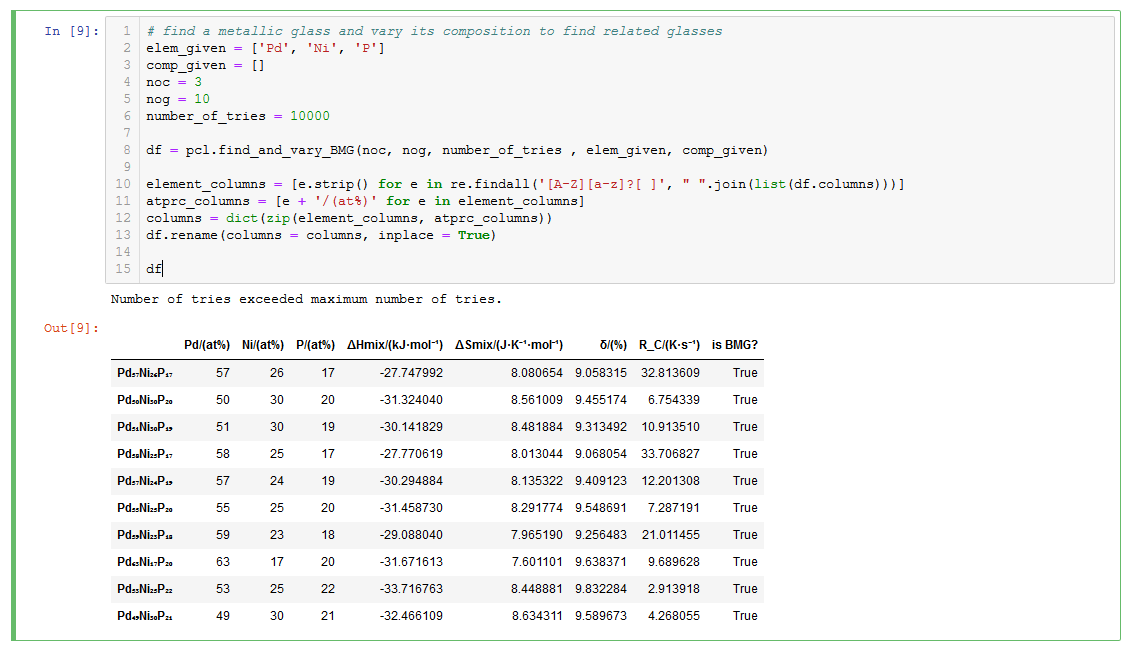

Supplement: Supplementary file 1 [file entropy-22-00292-s001.zip › QuickGuide/Step5.png]

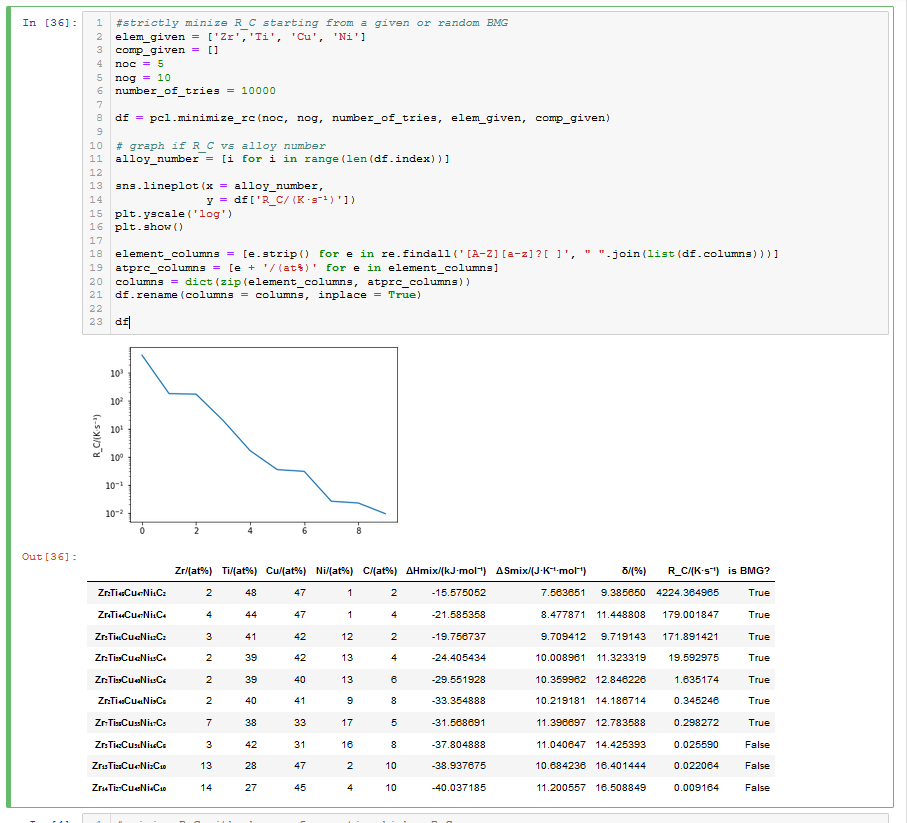

Supplement: Supplementary file 1 [file entropy-22-00292-s001.zip › QuickGuide/Step6.png]

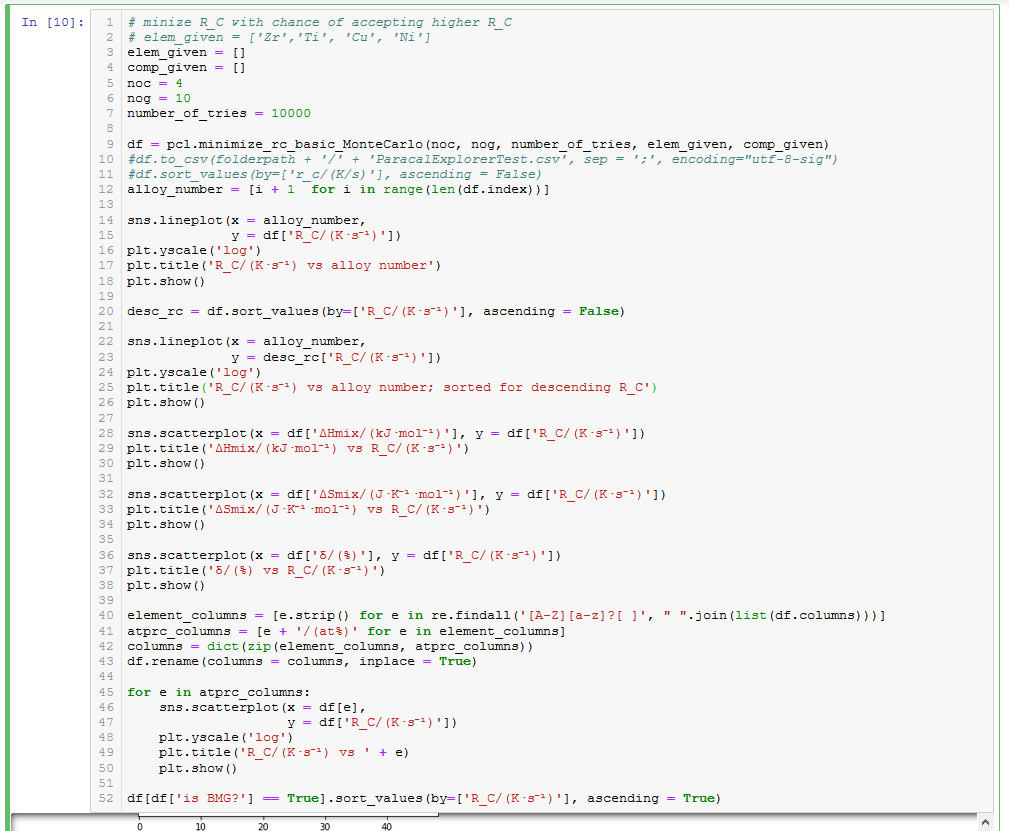

Supplement: Supplementary file 1 [file entropy-22-00292-s001.zip › QuickGuide/Step7.png]

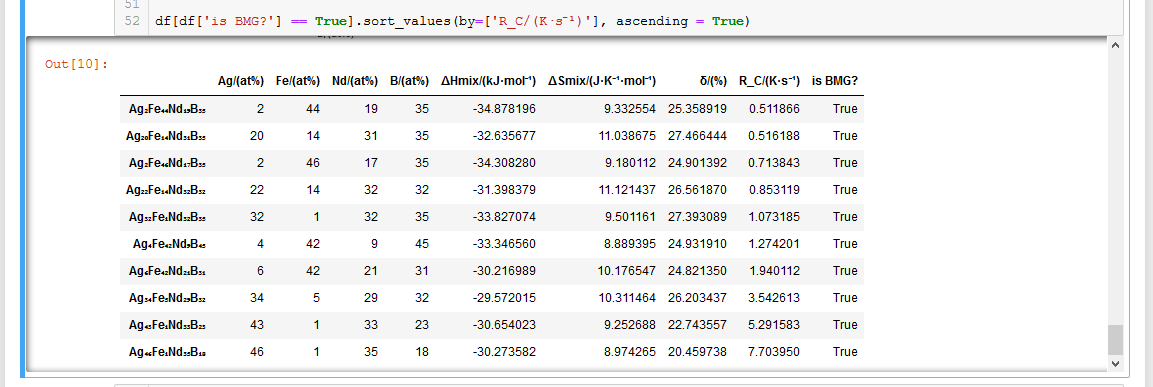

Supplement: Supplementary file 1 [file entropy-22-00292-s001.zip › QuickGuide/Step8.png]

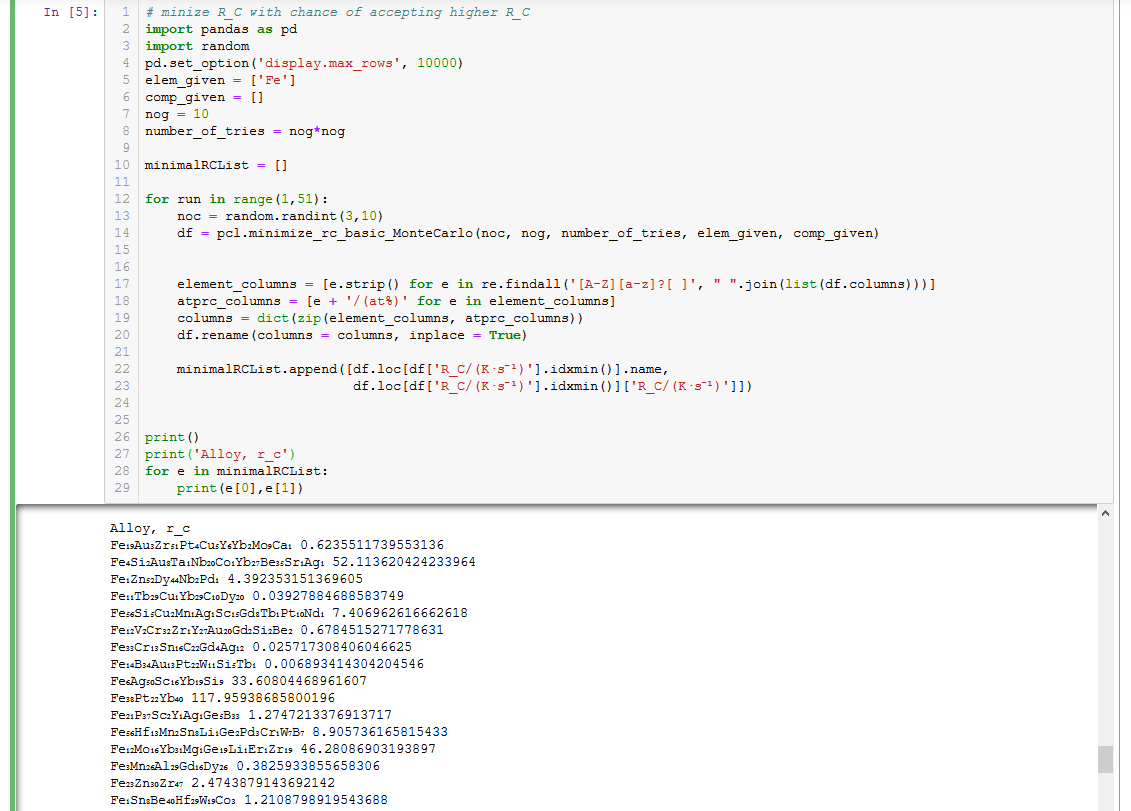

Supplement: Supplementary file 1 [file entropy-22-00292-s001.zip › QuickGuide/Step9.png]
